# Theoretical investigation on the ground state properties of the hexaamminecobalt(iii) and nitro–nitrito linkage isomerism in pentaamminecobalt(iii) *in vacuo*†

**DOI:** 10.1039/c7ra11603a

**Published:** 2018-01-16

**Authors:** Jules Tshishimbi Muya, Hoeil Chung, Sang Uck Lee

**Affiliations:** Hanyang University, Department of Chemistry Seoul South Korea hoeil@hanyang.ac.kr julescmuya.tmuya@gmail.com; Hanyang University, Department of Chemical & Molecular Engineering Sangnok-gu Ansan 426-791 Korea sulee@hanyang.ac.k; Hanyang University, Department of Bionanotechnology Sangnok-gu Ansan 426-791 Korea

## Abstract

Nitro–nitrito isomerization in Co(NH_3_)_5_NO_2_^2+^ linkage isomers was investigated with a focus on the geometries, relative stabilities and chemical bonding using ωB97XD/6-31+G(d,p) to elucidate the photo-salient effect in [Co(NH_3_)_5_NO_2_]NO_3_Cl. Different techniques like atoms in molecules (AIM), electron localization function (ELF) and natural bonding orbital (NBO) were used to gain insight into the chemical bonds of the isomers and to identify the key factors influencing their relative stabilities. The study of the ground-state potential energy surface of [Co(NH_3_)_5_NO_2_]^2+^ reveals that the nitro/*exo*-nitrito isomerization reaction can proceed *via* the following two paths: (1) nitro → TS1 (38.16 kcal mol^−1^) → *endo*-nitrito → TS2 (9.68 kcal mol^−1^) → *exo*-nitrito and (2) nitro → TS3 (41.76 kcal mol^−1^) → *exo*-nitrito. Pathway (1) through *endo*-nitrito is the most likely isomerization mechanism because of a lower energy barrier than pathway (2). The intramolecular-resonance-assisted hydrogen bonds (N–H⋯O and N–H⋯N), the orientation of NO_2_, and the difference between Co–N and Co–O bond energies are identified as the key factors determining the relative stabilities of the linkage isomers. Co(NH_3_)_6_^3+^ is less stable compared to Co(NH_3_)_5_NO_2_^2+^ and undergoes a slight geometrical distortion from *D*_3d_ to either *D*_3_ or *S*_6_ characterized by a stabilization energy of ∼750 cm^−1^ at CCSD(T)/6-31+G(d,p).

## Abstract 2

Nitro–nitrito isomerization in Co(NH_3_)_5_NO_2_^2+^ linkage isomers was investigated with a focus on the geometries, relative stabilities and chemical bonding using ωB97XD/6-31+G(d,p) to elucidate the photo-salient effect in [Co(NH_3_)_5_NO_2_]NO_3_Cl.

## Graphical abstract

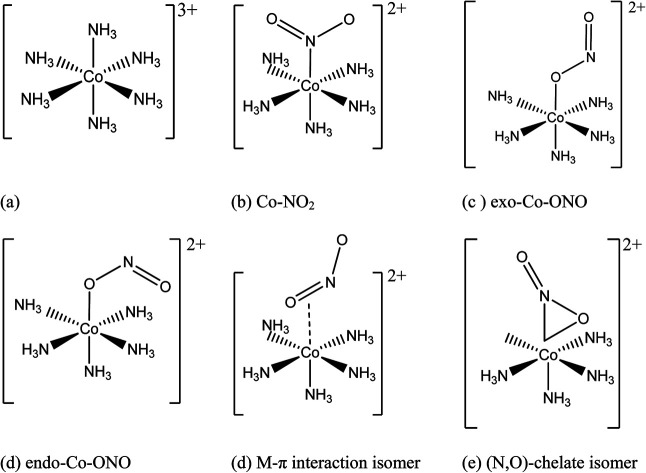

## 1. Introduction

Co complexes play various important roles in the chemistry of life processes and have been known for ages to impart blue color to ceramics.^1,2^ Considerable research effort has been made to understand the properties of Co complexes, particularly those derived from cobaltammines. Sakiyama *et al.*^3^ analyzed the electronic spectra of the hexaamminecobalt(iii) complex cation in aqueous solution to obtain the spectral components attributed to the slight distortion from a regular octahedron around the central cobalt(iii) ion and reported that the LC-BLYP/6-31G(d) optimized geometry of the complex in aqueous solution is a trigonally compressed octahedron under *D*_3d_. The influence of this reduction in the symmetry of [Co(NH_3_)_6_]^3+^ on its vibrational spectrum was also examined in the solid state.^4^ This trigonal deformation was reported to be sensitive to the environment of the complex.^3^ A crystallographic study of the binding of oxo-anions with cationic cobaltammine carried out by Sharma *et al.*^5^ revealed the presence of discrete [Co(NH_3_)_6_]^3+^ ions and mixed anions (*e.g.* Cl^−^ and ClO_3_^−^, Br^−^ and ClO_3_^−^, Cl^−^ and ClO_3_^−^) which are stabilized by hydrogen bonding interactions and attractive electrostatic forces. The hexaamminecobalt(iii) complex cation is a potential anion receptor^5,6^ widely used in structural biology to characterize biomolecules like DNA, RNA, and proteins^7^ and it is considered as a representative cationic Werner complex. It is worth mentioning that the works of Alfred Werner on cobaltammines led to a Nobel Prize in 1913,^8^ which formed the basis of modern transition-metal coordination complex chemistry, especially the linkage of atoms in transition metal compounds.

**Fig. 1:**
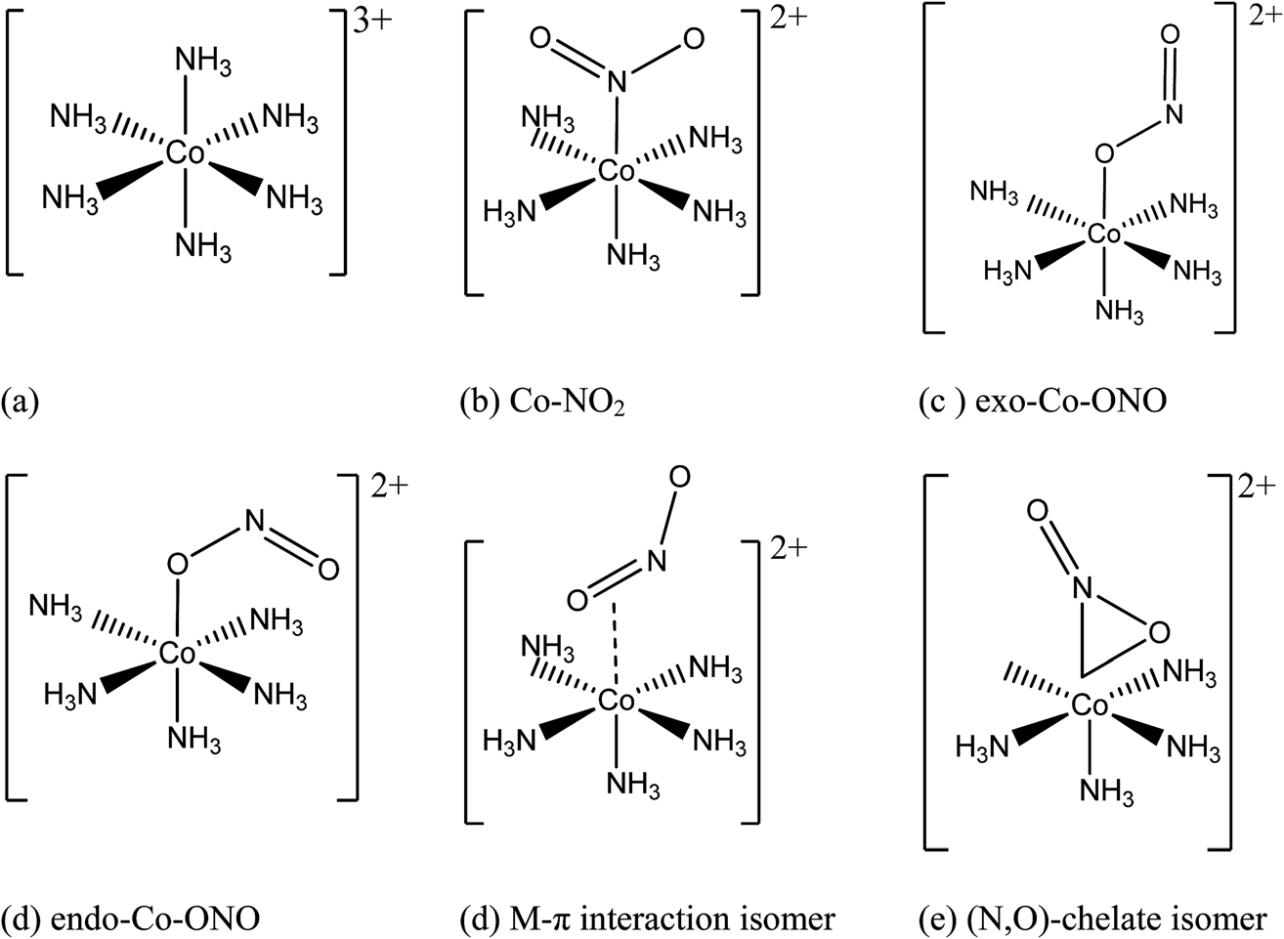
Initial geometries of: (a) hexaamminocobalt(iii) cation, (b–c) *N*- and *O*-linked Co-complexes isomers, (d) M–π interaction Co-complex isomer, and (e) (*N*,*O*)-chelate Co-complex isomer.

Transition-metal linkage isomers have the same chemical composition, differing only in the nature of the metal–ligand connectivity.^9^ For example, when one ammonia in hexaamminecobalt(iii) is substituted by a nitrite anion, nitro, *endo*-nitrito, and *exo*-nitrito isomers are formed (Fig. 1). Nitrite anion is an ambident ligand with electronic density delocalized on both N–O bonds, and both N and O atoms acting as alternative reactive sites. It can bind to the metal through N as well as through O, leading to linkage isomerism. The ambident reactivity of the nitrite ion was studied in detail by Tishkov *et al.*^10^ For instance, the cationic cobalt complex of formula [Co(NH_3_)_5_NO_2_]Cl_2_ exists as either the yellow-colored nitro isomer ([Co(NH_3_)_5_NO_2_]Cl_2_), where the nitro ligand is bound to Co through nitrogen, or the red-colored nitrito isomer ([Co(NH_3_)_5_ONO]Cl_2_), where the nitrito is bound to Co through one of the oxygen atoms^11–16^ Several research groups have focused their studies on metal linkage compounds due to their potential applications in medicinal therapy, photo-responsive materials, tunable optical materials, and molecular devices.^11–13,17–26^

The transformation of the N-bonded nitro isomer to the less stable O-bonded nitrito isomer has already been extensively studied experimentally and the conversion was demonstrated to occur intramolecularly.^13–16^ The nitrito isomers of [Co(NH_3_)_5_ONO](NO_3_)_2_ and [Co(NH_3_)_5_ONO](NO_3_)Cl convert slowly to the respective nitro isomers [Co(NH_3_)_5_NO_2_](NO_3_)_2_ and [Co(NH_3_)_5_NO_2_](NO_3_)Cl when placed in a dark room.^19^ The base dependence of this linkage isomerization and the transformation of the nitrito in nitrito was also observed overnight by Jackson *et al.*^27^ in [Co(NH_3_)_5_ONO](ClO_4_) in solution using H^1^ NMR and UV-vis techniques. The reaction of the transformation of [Co(NH_3_)_5_ONO]^2+^ in [Co(NH_3_)_5_NO_2_]^2+^ in solution and in solid state was found to be accelerated by light.^27–29^ More detailed information related to the experiments describing the synthesis of [Co(NH_3_)_5_NO_2_]^2+^ and [Co(NH_3_)_5_ONO]^2+^ and the application of spectroscopic techniques to detect the nitrite bonding mode are provided elsewhere.^27,29–32^ Here we only recall that this transformation may pass through an intermediate *endo*-[Co(NH_3_)_5_ONO]^2+^. According to Ciofini,^33^ experimental data do not provide a better understanding of the nitro/nitrito transformation mechanism, and theoretical methods can help in the elucidation of this reaction mechanism. This solid-state intramolecular reaction changes the configuration of the [Co(NH_3_)_5_NO_2_]^2+^ complex cation alone at its site in the lattice, leaving the rest of the lattice unchanged^9^ and was reported to be autocatalytic or auto-inhibitory.^24^ In contrast to the dark condition, Naumov *et al.*^22,23^ reported that the [Co(NH_3_)_5_NO_2_](NO_3_)Cl crystal exhibits a forceful jump when exposed to UV light (referred to as the photo-salient effect). During this photo-isomerization process, [Co(NH_3_)_5_ONO]^2+^ was thought to serve as a local source of strains.^24,25^

The discovery of photo-induced leaping in [Co(NH_3_)_5_ONO](NO_3_)Cl crystals stimulated extensive research into its structural and photo-rearrangement properties.^22,23,34,35^ The design of dynamic molecular crystals with properties that can be controlled by applying an external stimulus is an important challenge in molecular materials science. Herein we investigate in detail the geometry, relative stability, and chemical bonding in the ground state of the hexaamminecobalt(iii) cation and the nitro-/nitrito-pentaamminecobalt(iii) linkage isomers (denoted as Co–NO_2_ and Co–ONO) *in vacuo* using ωB97XD/6-31+G(d,p). Knowledge of the structural and electronic properties of the nitro-/nitrito-pentaamminecobalt(iii) linkage isomers is critical in the research of novel linked isomers with switchable optical properties. The main purpose of this study is the prediction of the pathway of the nitro-/nitrito-pentaamminecobalt(iii) isomerization because the latter is the probable cause of the photo-salient effect in [Co(NH_3_)_5_ONO](NO_3_)Cl.

This study is organized as follows:

(1) First, the relative stability of Co(NH_3_)_6_^3+^ having *D*_3d_ symmetry in *vacuo* is analyzed using group theory and various quantum chemical methods. Contrary to literature reports, we found [Co(NH_3_)_6_]^3+^ to be unstable in *D*_3d_ (possessing 6 imaginary frequencies) and it tends to distort towards either *D*_3_ or *S*_6_. [Co(NH_3_)_6_]^3+^ is compared to its analogous complexes *e.g.* [Ru(NH_3_)_6_]^3+^, [Cr(NH_3_)_6_]^3+^ and [Fe(NH_3_)_6_]^2+^ at B3LYP/LanL2DZ. This *D*_3_ geometry was afterwards used in an isodesmic reaction to compute the formation energies of [Co(NH_3_)_5_NO_2_]^2+^ isomers.

(2) Secondly, MP2, CCSD, B3LYP, B3LYP-D3, M062X, LC-BLYP, and ωB97XD methods are compared with CCSD(T). The ωB97XD and LC-BLYP methods gave good results approaching CCSD(T). Therefore, the subsequent analysis is performed using ωB97XD.

(3) Thirdly, the thermodynamics and kinetics of the Co(NH_3_)_5_NO_2_^2+^ isomers are studied at the ωB97XD level, and two nitro/*exo*-nitrito isomerization reaction paths are identified: (1) nitro → TS1 (38.16 kcal mol^−1^) → *endo*-nitrito → TS2 (9.68 kcal mol^−1^) → *exo*-nitrito and (2) nitro → TS3 (41.76 kcal mol^−1^) → *exo*-nitrito. The most likely reaction is the one that follows pathway (1) because it is the lowest-energy path.

(4) Lastly, the nature of chemical bonding in the nitro- and nitrito linkage isomers is examined briefly to understand how atoms are held together and to identify key factors to justify their order of stabilities. Hydrogen bonds, orientation of atoms in ONO group, and difference in natural bonding orbital (NBO) energy between Co–N and Co–O bonds are identified as the key factors to explain the relative stabilities of linkage isomers.

We confirmed through this analysis that the reaction path (1) through *endo*-nitrito is the most likely isomerization mechanism causing photo-salient effect, because it is the lowest energy reaction path.

## 2. Computational details

Fig. 1 shows the initial geometric structures used herein for geometry optimization. The [Co(NH_3_)_5_NO_2_]^2+^ linkage isomers were constructed following the coordination modes proposed in literature.^36,37^Fig. 1c and d display two O-bonded nitrito isomers with different spatial orientation of the atoms of the ONO group, denoted as *exo*-Co–ONO and *endo*-Co–ONO. Only singlet states were considered in the present study because octahedral Co(iii) prefers the low-spin state, contrary to the Co(ii) state.^38–40^ Comprehensive understanding of [Co(NH_3_)_6_]^3+^ and [Co(NH_3_)_5_NO_2_]^2+^ isomers requires state-of-the-art quantum chemical methods. All geometries were fully optimized and minima characterized by real vibrational modes at ωB97XD/6-31+G(d,p). Transition states were located using QST3 and IRC methods, followed by vibrational frequency analysis. All the minima energy values reported here are zero-point energy (ZPE) corrected using the expression:
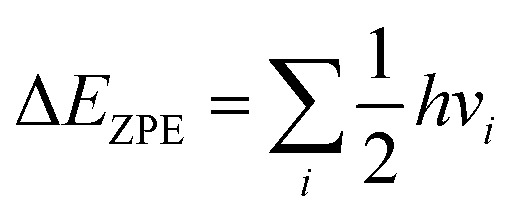
where *h* and *ν*_*i*_ stand for Plank's constant and the vibrational frequency of mode *Q*_*i*_, respectively.

CCSD(T) single-point calculations were performed based on CCSD optimized geometries to validate our methodology, and the structures and their relative energies were found to be similar to those obtained at ωB97XD. The dynamic correlation in *D*_3d_–Co(NH_3_)_6_^3+^ was traited on the top of the CASSCF(2,2) method with the Multireference Averaged Coupled-Pair Functional (MRACPF) approach to evaluate the Pseudo-Jahn–Teller stabilization energy (PJTE). The dispersion energies in the complexes were evaluated as an electronic energy difference between B3LYP and B3LYP-D3. The latter is composed of three terms: Kohn–Sham B3LYP, pair-wise London dispersion and three-body dispersion energies. Several studies^41,42^ have indicated that transition metals can have important relativistic effects that require detailed research. The zero-order regularized approximation to the Dirac equation (ZORA)^42^ was employed to treat relativistic effects. The symmetry disruption in the *D*_3d_ [Co(NH_3_)_6_]^3+^ was investigated using quantum mechanical calculations and group theory.^43^ Further, the *D*_3d_ epikernels of [Co(NH_3_)_6_]^3+^ were compared with [Ru(NH_3_)_6_]^3+^, [Cr(NH_3_)_6_]^3+^ and [Fe(NH_3_)_6_]^2+^ epikernels at B3LYP/LanL2DZ. The accumulation of electron density at the bond critical point (BCP),^44^ electron localization function (ELF),^45,46^ and natural bond orbitals (NBO)^47,48^ were undertaken to obtain useful information on the electron density charge distribution and for the rationalization of chemical bonding. Most of the optimization calculations were performed using Gaussian 09^49^ for single reference methods, whereas orca^50^ program was employed for treatment of multireference and relativistic effects. Further optimization of the epikernels geometries of [Co(NH_3_)_6_]^3+^ (*D*_3d_, *D*_3_, *S*_6_, *C*_2h_) were carried out using B3LYP/TZP on TURBOMOLE^51^ and SVWN/TZP method including ZORA on Amsterdam Density Functional (ADF).^52^ Gaussview 5.0,^53^ chemcraft,^54^ Jmol 13.0,^55^ were used for visualization while AIM2000,^56^ and Multiwfn^57^ programs were employed to analyze the topology of the chemical bonding in nitro/nitrito linkage isomers.

## 3. Results and discussion

### 3.1 [Co(NH_3_)_6_]^3+^

#### 3.1.1 Analysis of the structural distortion from *D*_3d_ to *D*_3_ symmetry

**Fig. 2:**
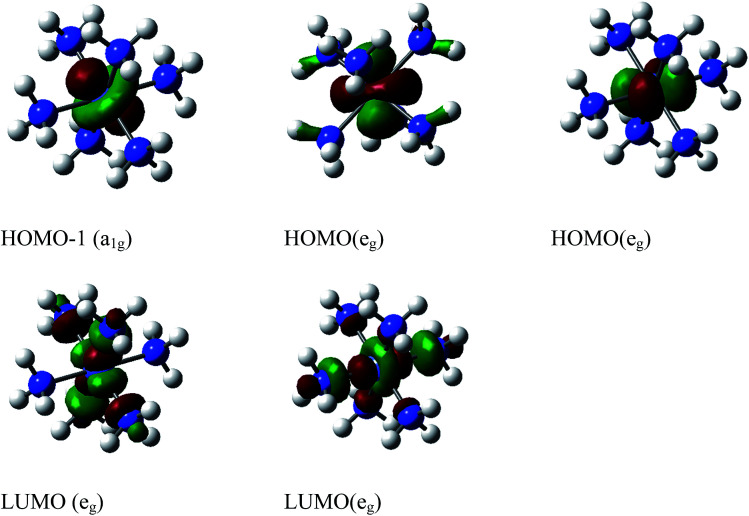
The Frontier molecular orbitals of *D*_3d_–[Co(NH_3_)_6_]^3+^ computed at ωB97XD/6-31+G(d,p).

In *D*_3d_–[Co(NH_3_)_6_]^3+^ the *t*_2u_ (*t*_2g_) orbital from octahedral symmetry (*O*_h_) splits in *e*_u_ and *a*_2u_ (*e*_g_ and *a*_2g_). The Frontier molecular orbitals of *D*_3d_–[Co(NH_3_)_6_]^3+^ are shown in Fig. 2, and the energy levels of valence MOs are listed in Table S1.† The HOMO and LUMO in *D*_3d_–[Co(NH_3_)_6_]^3+^ are on ammonia and belong respectively to *e*_g_ and *a*_1g_ representation. Their energy difference computed is nearly 11.11 eV at ωB97XD, 13.25 eV at LC-BLYP and 17.74 eV at MP2 (17.70 eV at CCSD(T)/6-31+G(d,p)). LC-BLYP, and ωB97XD methods have tendency to underestimate the HOMO–LUMO gaps compare to MP2 method. LC-BLYP, ωB97XD, and MP2 methods yield Co–N bonds distances of 1.97–2.01 Å close to CCSD and BD(T) bond lengths calculated about 1.99–2.00 Å in gas phase (Table 1). The experimental value of the equilibrium Co–N bond length of Co(NH_3_)_6_^3+^ is measured and found to be around 1.97 Å;^58–60^ this is not significantly different from CCSD/6-31+G(d,p) calculated bond in the gas phase. This small difference between experiment and calculated bond distance is explained by different environment of [Co(NH_3_)_6_]^3+^ in solid. It has been reported in solution that the neglect of solvent–solute charge transfer effect can yield too long Co–N bond lengths.^61^

**Table 1:** Co–N bonds in Å, HOMO–LUMO (H–L) gap energies in eV, lowest vibrational frequencies (LF) in cm^−1^ and PJT stabilization energies in kcal mol^−1^ of *D*_3_–[Co(NH_3_)_6_]^3+^ computed using different methods in conjunction with 6-31+G(d,p)

Methods	Co–N	PJT energy	H–L gaps	LF (symmetry)
BD(T)	2.001	2.16	17.24	—
CCSD(T)	1.992	2.14	17.74	—
MP2	1.973	2.43	17.79	—
B3LYP	2.013	1.75	6.28	75(e)
B3LYP-D3	2.004	1.77	6.24	78(e)
M062X	2.022	2.04	10.89	106(e)
ωB97XD	2.009	2.12	11.13	89(e)
LC-BLYP	1.976	2.14	13.27	83(e)
Exp. [ref. 55]	1.97			

Our calculations show that *D*_3d_*in vacuo* is unstable contrary to what was reported elsewhere,^3^ in solid and aqueous media. Its optimized geometry is characterized by six imaginary modes of representations *a*_1u_, *a*_2g_, *e*_g_ and *e*_u_ at i167, i164, i101 and i90 cm^−1^ at ωB97XD/6-31+G(d,p) (i154, i150, i74 and i45 cm^−1^ at CCSD/6-31G(d)), respectively. A distortion with the *D*_3_ symmetry constraint yields a minimum energy characterized by the lowest vibrational mode of *e* symmetry located at 89 cm^−1^ at ωB97XD/6-31+G(d,p) (79 cm^−1^ at CCSD/6-31G(d)). The energy difference between the HOMO and LUMO is slightly increased to 17.74 eV at CCSD(T)/6-31+G(d,p) for *D*_3_. A further optimization of *D*_3_ and *D*_3d_ geometries using B3LYP/TZP method as implemented in TURBOMOLE gives an energy difference of 1.54 kcal mol^−1^. The HOMO–LUMO gap in gas phase is 6.25 eV. The vibrational analysis is characterized by two imaginary frequency at i107.97 and i104.40 cm^−1^ of *a*_1u_ and *a*_2g_ representations, whereas a full optimization of the same geometry in *D*_3d_ at the SVWN/TZP including the scalar and spin–orbit ZORA as implemented in ADF program reproduces six imaginary frequencies estimated about i137.99 (*a*_1u_), i137.90 (*a*_2g_), i54.56 (*e*_g_), i18.00 (*e*_u_) cm^−1^ confirming that *D*_3d_ is not the true global minimum of [Co(NH_3_)_6_]^3+^.

The structural change between *D*_3d_ and *D*_3_ in [Co(NH_3_)_6_]^3+^ is small (Fig. S2†). The distortion vector responsible of this symmetry breaking can be expressed as:^62,63^1
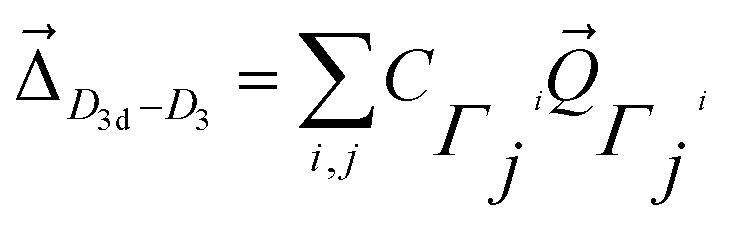
where the coefficient in the expression is related to the weight of the imaginary mode 
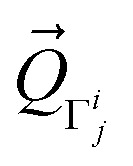
, and the subscript describes the irreducible representation of the vibrational mode.

Our calculations show a significant contribution of the 1*a*_1u_ imaginary mode of nearly 98.89% in the distortion vector (Fig. S3†). A further distortion of *D*_3d_ along the imaginary vibrational mode *a*_2g_ will lead to *S*_6_. The distortion of *D*_3d_–[Co(NH_3_)_6_]^3+^ along 1*a*_1u_ vibrational mode acts essentially on H and destroys the center of symmetry. The full *D*_3d_ symmetry group is broken because by destroying the inversion point, 1*a*_1u_ will also remove the improper rotation axis (*S*_6_) and the dihedral plane. However, the imaginary modes show a rotation of hydrogen atoms. The free rotation of NH_3_ groups in octahedral symmetry observed in water solvent should not strictly be applicable to low symmetry complex. The decent in symmetry observed from *D*_3d_ to its subgroup *D*_3_ can only arise from the Pseudo-Jahn–Teller (PJT) effect because the HOMO (*e*_g_)^4^ in *D*_3d_ is fully occupied and cannot trigger a pure Jahn–Teller distortion according to group theory. The computed PJT energy caused by this vibronic coupling is about 742 cm^−1^ at ωB97XD/6-31+G(d,p) (∼750 cm^−1^ at CCSD(T)/6-31+G(d,p)). The Pseudo-Jahn–Teller computed at CASSCF(2,2)-MRCAPF/SVP is about 154 cm^−1^ at SVP. Krisloff *et al.*^64^ investigated the unphysical ground states of the multireference averaged coupled-pair functional and reported that CASSCF(2,2)-MRCAPF can provide unphysical solution in the avoided crossing or conical intersection and the instabilities can be removed by employing larger complete active spaces (CAS). Unfortunately, the latter is computationally more demanding. The molecular orbitals involved in this distortion mechanism require further investigation. The energy level of the valence molecular orbitals of the *D*_3d_ geometry is listed in Table S1† and agree with the literature.^65^ The HOMOs are localized on the metal and are nonbonding pure d orbitals. The LUMOs have high contribution on the metal and nitrogen and are antibonding orbitals (Fig. S1†). The HOMO (*e*_g_) and HOMO-1 (*a*_1g_) derived from the reduction of *t*_2g_ in *O*_h_. To validate the use of ωB97XD, various DFT and MP2 methods were employed and compared with CCSD(T). The data obtained shown in Table 1 suggest ωB97XD and LC-BLYP could perform quite well with respect to CCSD(T). Therefore, the ωB97XD/6-31+G(d,p) method and the lowest *D*_3_ geometry are considered for further analysis on nitro-/nitrito-pentaamminecobalt(iii) linkage isomers.

We have extended this study to other hexammines complexes analogs of Ru(iii), Cr(iii) and Fe(ii) which were reported to have either *D*_3d_ or *D*_3_ symmetries.^3,58,65–74^ [Ru(NH_3_)_6_]^3+^, [Cr(NH_3_)_6_]^3+^ and [Fe(NH_3_)_6_]^2+^ ions can be considered as octahedral in which the d orbital of the transition metal splits in *t*_2g_ and *e*_g_. For comparison, the most popular functional for transition metal B3LYP was employed with LanL2DZ basis set for all atoms on the *D*_3d_ symmetry of the low-spin states [^1^*A*_1g_: (*t*_2g_)^6^ (*e*_g_)^0^ ] and [^2^*T*_2g_: (*t*_2g_)^5^ (*e*_g_)^0^] of [Co(NH_3_)_6_]^3+^ and [Ru(NH_3_)_6_]^3+^_,_ and high spin states [^4^*T*_2g_: (*t*_2g_)^3^ (*e*_g_)^0^] and [^5^*T*_2g_: (*t*_2g_)^4^ (*e*_g_)^2^] of [Cr(NH_3_)_6_]^3+^ and [Fe(NH_3_)_6_]^2+^. The Pseudo-Jahn–Teller energies obtained at B3LYP/LanL2DZ for the symmetry breaking of *D*_3d_ towards either *D*_3_ or *S*_6_ are given in Table 2. *D*_3_ and *S*_6_ are higher ranking epikernels and are found as minima for all transition metal complexes studied in the present paper, whereas the lower ranking epikernel *C*_2h_ is found to be a saddle point [Tables 2 and 3]. The difference in metal-N bonds between *D*_3_ and *S*_6_ is negligible (∼0.001 A). These findings are in good agreement with the epikernel principle.^63^ The Pseudo-Jahn–Teller distortion energies are considerably more important in Co- and Cr-complexes than Ru- and Fe-complexes. The M–N bonds distances listed in Table 2 computed at B3LYP/LanL2DZ are close to that obtained at high level of calculations.^61,67–71^ For instance, the Co–N bond evaluated about 2.037 at B3LYP/LanL2DZ is approaching that obtained by Rotzinger^61^ at CASSF-MP2(10,10)/6-31G(d) and CASSF(10,10)/6-31G(d) levels, estimated about 2.014 and 2.034. The *D*_3_ [Fe(NH_3_)_6_]^2+^ geometry was also predicted as minima by Pierloot *et al.*^70^ in their study on the performance of BP86, B3LYP and PBE0 with CASPT2 in the estimation of the relative energy of the high and low spins states of [Fe(H_2_O)_6_]^2+^, [Fe(NH_3_)_6_]^2+^ and [Fe(bpy)_3_]^2+^. According to the authors, DFT even in cases it does not provide accurate energetics, still provides high quality structure.

**Table 2:** M–N distances (with M transition metal), pseudo-Jahn–Teller energies (cm^−1^) and lowest real frequencies computed at B3LYP/LanL2DZ of *D*_3_ and *S*_6_ in parenthesis of [Co(NH_3_)_6_]^3+^, [Ru(NH_3_)_6_]^3+^, [Cr(NH_3_)_6_]^3+^ and [Fe(NH_3_)_6_]^2+^ at B3LYP/LanL2DZ

	State	M–N	Ref.	PJTE	LF
Co	^1^A_1g_	2.037	2.014,^61^ 2034,^61^ 2.009,^67^ 1.967^58^	749 (596)	94 (48)
Ru	^2^A_1g_	2.193	2.104,^68^ 2.113^67^	209 (237)	56 (56)
Cr	^4^A_1g_	2.149	2.060^69^	594 (463)	75 (55)
Fe	^5^A_1g_	2.295	2.293,^70^ 2.21^71^	167 (101)	38 (41)

**Table 3:** Number and symmetry of imaginary modes in *C*_2h_ and *D*_3d_ optimized geometries computed at B3LYP/LanL2dz and relative energies in kcal mol^−1^ with respect to *D*_3d_ geometry

State in *D*_3d_	*C*_2h_	*D*_3d_	RE
Co[^1^A_1g_]	i70(a_u_), i50(b_g_)	i160(a_1u_), i155(a_2g_), i77(e_g_), i36(e_u_)	−1.60
Ru[^2^A_1g_]	i52(a_u_), i48(b_g_)	i106(a_2g_), i98(a_1u_), i23(e_g_)	0.05
Cr[^4^A_1g_]	i140(a_u_), i114(b_g_), i92(b_g_), i60(b_u_)	i138(a_1u_), i134(a_2g_), i69(e_g_), i43(e_u_)	−0.13
Fe[^5^A_1g_]	i51(e_u_),	i80(a_1u_), i67(a_2g_)	−0.33

Contrary to *D*_3_ symmetry which symmetry was mentioned by many researchers,^61,70^*S*_6_ symmetry is proposed in the present paper for the first time as probable minima for [Co(NH_3_)_6_]^3+^, [Cr(NH_3_)_6_]^3+^, [Ru(NH_3_)_6_]^3+^ and [Fe(NH_3_)_6_]^2+^ in gas phase. Tables S2 and S3† compare B3LYP, ωB97XD and OPBE results of [Co(NH)_3_]_6_^3+^ computed with ccpVtz basis sets to check the basis set dependency. The OPBE functional was suggested by Swart *et al.*^72^ to provide accurate geometries for several transition metal compounds. The relative energies of *D*_3_, *S*_6_ and *C*_2h_ with respect to *D*_3d_ computed with OPBE method lay in between ωB97XD and B3LYP. *D*_3_ is slightly more stable than *S*_6_ at different levels of calculations. The EOMCCSD/6-31+G(d,p) calculations from *D*_3_ optimized geometry show three bands at 114, 129, and 162 nm assigned to respectively ligand to ligand transition (4*t*_1u_ → 5*a*_1g_), and ligand to metal charge transfer transitions (1*a*_1u_ → 5*e*_g_ and 4*t*_1u_ → 5*e*_g_) which lie close to the greatest absorbance experimental value of about 195 nm reported by Goursot *et al.*^65^ They argue that the *ab initio* energy values of these charge transfer transitions are largely dependent on the basis set chosen. A similar transition was observed in [Ru(NH_3_)_6_]^3+^ UV-vis spectrum also arising from *t*_1u_ and *e*_g_.^73,74^ Daul *et al.*^74^ investigated the electronic structure of [Ru(NH_3_)_6_]^3+^ using extended Huckel molecular orbitals method and reported that ammonia are rotating freely to keep an octahedral site symmetry. The d–d transitions in octahedral [Co(NH_3_)_6_]^3+^ and [Ru(NH_3_)_6_]^3+^ complexes are forbidden due to the presence of a center of inversion following Laporte rule^75^ which states that allowed transitions in centro-symmetric molecules should involve change in parity between gerade and ungerade orbitals. In real world, [Co(NH_3_)_6_]^3+^ and [Ru(NH_3_)_6_]^3+^ geometries are not frozen but thermally vibrating. The oscillator strength of the symmetry-forbidden d–d transition of the octahedral transition metal complexes, [Co(NH_3_)_6_]^3+^ and [Ru(NH_3_)_6_]^3+^, was evaluated theoretically by Saito *et al.*^67^ using B3PW91 functional with the effective core potential/basis set combination SDB-cc-pVTZ. The authors found that the oscillator strengths of ^1^*A*_1_ → ^1^*T*_1g_ is considerably larger than that of the ^1^*A*_1g_ → ^1^*T*_2g_ in both complexes and arises from H_3_N–M–NH_3_ antisymmetric bending vibration. The metal ligand charge transfer (MLCT) was found larger in the Ru-complex than in its counterpart Co-complex. Multiconfigurational self-consistent-field (MCSCF) approaches are more efficient way one can use for diagnosticate the pseudo-Janh–Teller effect in *D*_3d_–[M(NH_3_)_6_]^3+^ (with M = Co, Fe, Cr, Ru).^76^ Symmetry plays a crucial role in Physics and Chemistry because the properties of molecular systems under symmetry transformation govern selection rules for molecular spectroscopy and interactions.^77^

### 3.2 [Co(NH_3_)_5_NO_2_]^2+^ and [Co(NH_3_)_5_ONO]^2+^

#### 3.2.1 Geometries

##### Geometries of [Co–NO_2_]^2+^ and [Co–ONO]^2+^ isomer compared to *D*_3_–[Co(NH_3_)_6_]^3+^

**Fig. 3:**
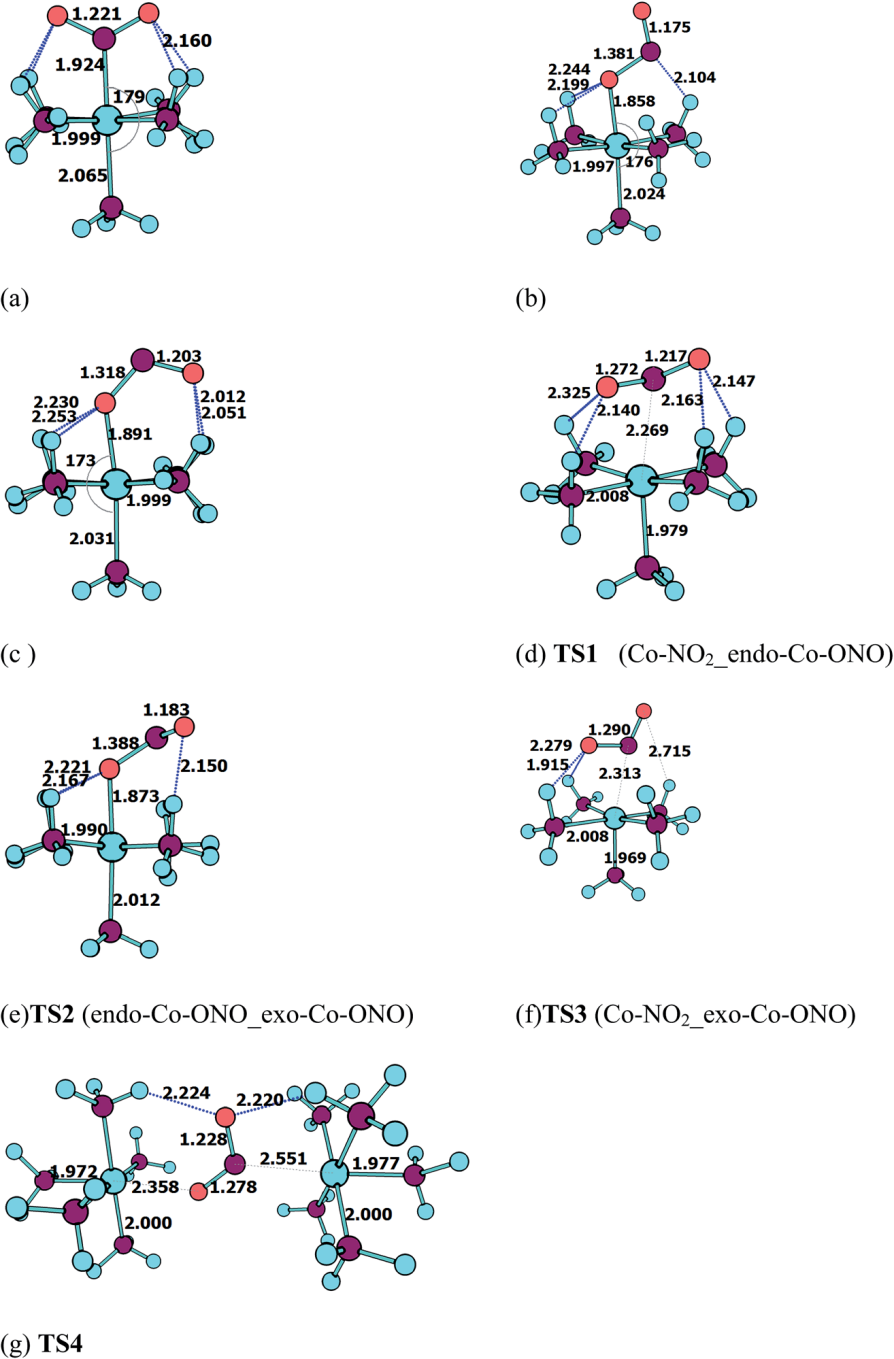
Equilibrium geometries (a–c) and transition states geometries (d–g) of Co(NH_3_)_5_NO_2_^2+^ and Co(NH_3_)_5_ONO^2+^ isomers computed at ωB97XD/6-31+G(d,p).

Fig. 3 shows the geometries of the ground states of *O*- and *N*-linked isomers and transition states computed at ωB97XD/6-31+G(d,p). A close inspection on the geometric structures shows that the substitution of ammonia by nitro or nitrito in [Co(NH_3_)_6_]^3+^ to form [Co(NH_3_)_5_NO_2_]^2+^ or [Co(NH_3_)_5_ONO_2_]^2+^ gives rise to the shrinking of Co–N bonds formed by NH_3_ positioned in the equatorial region with the concomitant elongation of the axial Co–NH_3_ bond. The NH_3_ ligands in the horizontal plane are apparently more strongly bound to Co than the axial NH_3_ ligands. In Co–NO_2_ (Fig. 3a), the bond length between Co and NO_2_ is 1.924 Å, which is 0.075 and 0.141 Å shorter than those in the Co–N bonds formed by equatorial and axial NH_3_. The Co–O bond length in the *exo*-ONO complex (Fig. 3b) is ∼0.03 Å shorter than that of the *endo*-ONO complex (Fig. 3c). The Co–NO_2_ and *exo*-Co–ONO geometries were also studied by Ciofini *et al.*^33^ at B3LYP/LanL2DZ level in order to clarify the intrinsic and environmental effects on the kinetic and thermodynamics of linkage isomerizasion in nitropentaamminecobalt(iii) complex. The geometrical parameters of our Co–NO_2_ and Co–ONO complexes optimized at ωB97XD/6-31+G(d,p) are compared in Table S4† with B3LYP/Lanl2dz geometries obtained by Ciofini *et al.*^33^ and the available X-ray data.^78,79^ ωB97XD/6-31+G(d,p) performs better than B3LYP/Lanl2dz and the small difference observed between X-ray and the optimized geometries can be attributed to crystal environment.^33^ Boldyreva *et al.*^80^ found different geometrical parameters in the pentaamminenitrocobalt(iii)chloride nitrate at 298 K and 150 K using X-ray spectroscopy characterized by Co–N, and N–O distances in the range of 1.959–1.989 and 1.206–1.222, and O–N–O and O–N–Co bonds angles of 123.1 and 117.8 degrees.

All these ωB97XD/6-31+G(d,p) optimized geometries comprise intramolecular hydrogen bonding (IHB) involving NO_2_ or ONO. The Co–NO_2_ complex has a O⋯H–N bond of 2.160 Å, which is 0.148 Å longer than the shortest O⋯H–N bond in the *endo*-Co–ONO complex. The *exo*-Co–ONO is characterized by two types of IHBs: N⋯H–N and O⋯H–N of 2.104 and 2.199–2.244 Å. The two N–O bonds that have the same bond lengths in the nitro-complex becomes unequal when the nitro is orientated in nitrito, and the difference between the bond lengths increases from the *endo*-nitrito to *exo*-nitrito complex, showing a decrease in the electron resonance character of the ONO atoms group. Electron delocalization around ONO was reported^81^ to enhance hydrogen bonds.

**Fig. 4:**
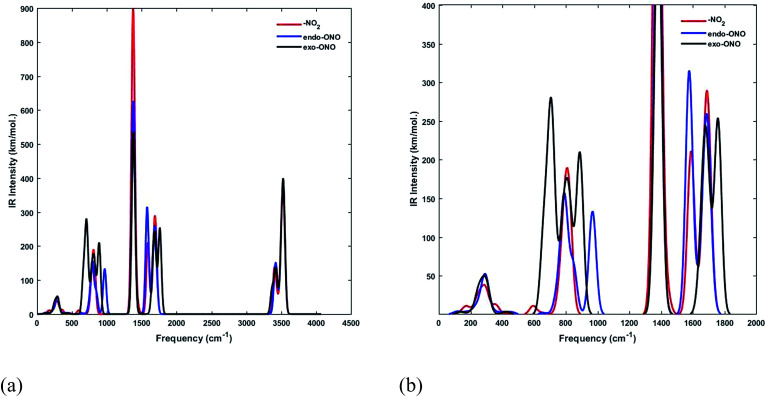
Superimposed IR spectra of Co–NO_2_ and Co–ONO complexes computed using a scaling factor of 0.9632 at B3LYP/6-31+G(d,p).

**Fig. 5:**
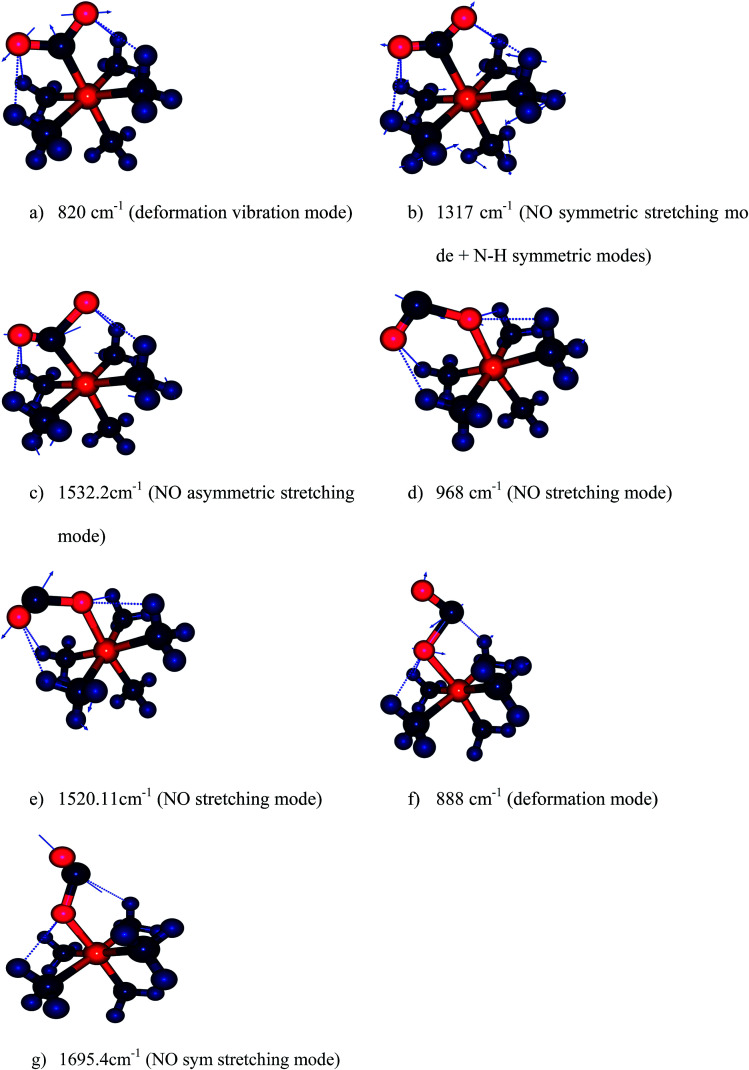
Harmonic vibration modes of NO_2_ and ONO in Co–NO_2_ (a–c), *endo*-Co–ONO (d–e) and *exo*-Co–ONO (f–g) computed at B3LYP/6-31+G(d,p).

We have computed the IR spectra of nitro and nitrito complexes at B3LYP/6-31+G(d,p). Our calculations show a clear difference in vibrational frequencies between NO_2_ and ONO isomers in the region between 700–1700 cm^−1^ (Fig. 4). The vibrational modes which characterize the NO_2_ and ONO in Co–NO_2_ and Co–ONO complexes are listed in Table 4 and shown in Fig. 5. The deformation NO_2_ and ONO modes are well reproduced whereas our asymmetric N–O stretching modes are overestimated referring to Ciofini findings and experimental data.^33^

**Table 4:** Harmonic vibrational frequencies (cm^−1^) computed at B3LYP/6-31+G(d,p) of Co–NO_2_ and Co–ONO isomers

Co–NO_2_	*Endo*-Co	*Exo*-Co–ONO	Assignment
820(757^a^, 825^b^)		880 (718^a^, 791^b^)	NO_2_ and ONO deformation
1367(1257^a^, 1315^b^)	968		Sym. NO_2_ stretching + NH_3_ defor.
1590(1466^a^, 1440^b^)	1578	1759(1586^a^, 1460^b^)	Asym. NO_2_ and ONO stretching

a Theoretical values taken from ref. 33a.

b Experimental data taken from ref. 33b.

##### Transition states

Co–NO_2_ → *endo*-Co–ONO → *exo*-Co–ONO reaction mechanism describes the sequence of the elementary transformation of Co–NO_2_ complex in *exo*-Co–ONO *via* the *endo*-Co–ONO complex. TS1 (i569 cm^−1^ computed at ωB97XD/6-31+G(d,p)) and TS2 (i210 cm^−1^ ωB97XD/6-31+G(d,p)) depicted in Fig. 3d and e are predicted sequential transition states in this reaction path by intrinsic reaction coordinate (IRC) method. TS1 has a NO_2_ moiety detached from Co; the Co–N bond length is estimated to be about 2.27 Å, but still binds to four NH_3_ ligands through O⋯H–N hydrogen bonds ranging between 2.14 and 2.32 Å at ωB97XD/6-31+G(d,p). The TS1 geometry was reported as first-order saddle point with one imaginary vibrational mode located at i385 cm^−1^at B3LYP/LanL2DZ.^33^ Its geometrical parameters geometry computed at ωB97XD/6-31+G(d,p) and B3LYP/LanL2DZ are detailed in Table S4.† This transition state is difficult to detect experimentally.^15^ The structural geometry of TS2 (Co–O bond of 1.87 Å) is similar to that of *exo*-Co–ONO but differs with *exo*-Co-ONO by the orientation of N–O bonds, which lies in the horizontal plane to form N–O⋯H hydrogen bonds of 2.15 Å with ammonia computed at ωB97XD/6-31+G(d,p). Another transition state similar to TS1, denoted as TS3 (i506 cm^−1^) (Fig. 3f) was observed between Co–NO_2_ and *exo*-Co-ONO and was characterized by two O⋯H–N hydrogen bonds of 1.91 and 2.28 Å carried out at ωB97XD/6-31+G(d,p). The N–O (1.29 E) and N–Co (2.31 E) bonds in TS3 are slightly longer than that of TS1. The π-bonding coordination to the central metal observed in these activated complexes (TS1, TS3) was also suggested by Balt *et al.*,^82^ but the authors did not provide the geometries of transition states. The imaginary vibrational modes of these transitions states are shown in Fig. S4.† The displacements of atoms displayed in Fig. S4† indicate that the imaginary modes of TS1 and TS2 change the Co–O–N–O torsion angle and O–N–O bond angle, whereas in TS3, the imaginary frequency attempts to rotate NO_2_ atoms group. The change in TS3 between Co–NO_2_ and *exo*-Co-ONO is mediated when the nitro atoms group rotates such that N or O can form the Co–N or Co–O bond, respectively. TS4 represented in Fig. 3g (i95 cm^−1^) was proposed in ref. 83 and 84 as the activated complex to explain the nitro and nitrito interconversion in [Co(NH_3_)_5_NO_2_]NO_3_Cl through the intermolecular process. In TS4, two groups of Co(NH_3_)_5_^3+^ are held together by the nitro and nitrito ligands through O⋯H–N hydrogen bonds of 2.220 and 2.224 Å, respectively.

##### Origin of mechanical motion in solids

The electronic spatial extents^85^ of [Co(NH_3_)_5_ONO_2_]^2+^ and [Co(NH_3_)_5_NO_2_]^2+^ were computed to gauge the increase in the size of those cations upon intramolecular conversion. The *exo*-Co-ONO has a larger electronic spatial extent than Co–NO_2_: these are estimated to be about 1556 and 1411 a.u., respectively (1451 for *endo*-Co–ONO). The effective size of the *exo*-Co-ONO isomer was evaluated to be 1.2 times greater than that of the NO_2_–Co.^16,17^ According to Boldyreva,^24^ the former is expected to play an important role in increasing the local pressure near the product in nitro and nitrito interconversion reaction in solids, and can be at the origin of the mechanical motion observed in crystals. Thus, it is important to first understand this isomerization reaction as this constitutes the fundamental process that triggers the photo-salient phenomenon in [Co(NH_3_)_5_NO_2_]NO_3_Cl.

#### 3.2.2 Thermodynamic and kinetic stability

##### Relative energy

Table 5 compares the relative energies of *exo*-Co-ONO and *endo*-Co-ONO complexes and their transitions states (TS1 and TS2) with respect to the Co–NO_2_ isomer at ωB97XD/6-31+G(d,p). According to the data listed in Table 5, the Co–NO_2_ complex is 1.32 and 3.39 kcal mol^−1^ lower than *endo*-Co-ONO and *exo*-Co-ONO complexes, respectively (1.12 and 2.00 kcal mol^−1^ at CCSD(T)/6-31G(d)) on the potential energy surface (PES). Noted that the relative energy of the *exo*-Co-ONO computed by Ciofini *et al.*^33^ is about 3.9 kcal mol^−1^ at B3LYP/LanL2DZ including ZPE corrections in gas phase. Thus, the nitro form is predicted to be the lowest isomer. The fact that the ωB97XD/6-31+G(d,p) energy ordering of isomers is reconfirmed by CCSD(T) (Table S5†) shows that the ωB97XD functional is an alternative method at an affordable cost for studying these linkage isomers. The dispersion energies in NO_2_–Co, *endo*-Co-ONO, and *exo*-Co-ONO isomers computed using dispersion-corrected B3LYP at 6-31+G(d,p) amount to −26.25, −25.43 and −24.95 kcal mol^−1^, respectively. The zero-order regular approximation (ZORA), a two-component form of the fully-relativistic Dirac equation used along with B3LYP at SVP, also indicates that Co–NO_2_ is the most stable isomer, followed by *endo*-Co-ONO. The difference in relative energies obtained using non-relativistic and relativistic method is small, ranging from 0.13 to 0.5 kcal mol^−1^.

**Table 5:** Relative electronic energies, enthalpies, free energies (in kcal mol^−1^), entropies (in cal mol^−1^) and HOMO–LUMO gap energies (in eV) of the ground states of different isomers and corresponding transition states computed at ωB97XD/6-31+G(d,p)

gs, TS	Δ*E*	Δ*H*	Δ*G*	Δ*S*	H–L
Co–NO_2_	0.00	0.00	0.00	0.00	8.68
*Endo*-Co–ONO	1.32	0.90	2.47	−5.25	7.92
*Exo*-Co–ONO	3.39	3.58	3.53	0.17	7.64
TS1	39.48	39.35	40.01	−2.22	—
TS2	11.00	10.95	11.00	−0.16	—
TS3	41.87	41.77	42.46	−2.30	—

##### Binding energy

The binding energies of NH_3_ and NO_2_^−^ to the metal in Co-complexes and the formation energy (FE) of complexes were obtained as electronic energy (*E*) difference between the most stable geometries of the products and reactants at ωB97XD/6-31+G(d,p) from the following equations:

2 Co(NH_3_)^3+^_5_ + NH_3_ → Co(NH_3_)^3+^_6_

3 Co(NH_3_)^3+^_5_ + NO^−^_2_ → Co(NH_3_)_5_NO^2+^_2_

4 Co(NH_3_)^3+^_6_ + NO^−^_2_ → Co(NH_3_)_5_NO^2+^_2_ + NH_3_

5 FE = *E*_(Co(NH_3_)_5_NO_2_^2+^)_ + *E*_(NH_3_)_ − *E*_(Co(NH_3_)_6_^3+^)_ − *E*_NO_2_^−^_

The heat of formation of Co(NH_3_)_5_NO_2_^2+^ at 298 K calculated by means of the isodesmic reaction (eqn (5)) is −280.55 kcal mol^−1^. The binding energies of NO_2_ and NH_3_ to the metal Co were found to be about −343.49 and −61.70 kcal mol^−1^, showing that NO_2_ binds more strongly to Co than NH_3_ does. Thus, the Co(NH_3_)_5_NO_2_^2+^ complex appears to be thermodynamically more stable than Co(NH_3_)_6_^3+^.

##### HOMO–LUMO & transition states

In conceptual DFT formalism, the HOMO–LUMO gap energy is related to the kinetic stability. The NO_2_–Co complex in Table 4 has the largest HOMO–LUMO gap of 8.68 eV, followed by *endo*-Co-ONO with a HOMO–LUMO gap energy of 7.92 eV. The HOMO–LUMO of *exo*-Co-ONO is 0.28 eV lower than that of *endo*-Co-ONO at ωB97XD/6-31+G(d,p). Therefore, by considering the HOMO–LUMO gap energy, Co–NO_2_ appears to be kinetically more stable than ONO–Co complexes. For this purpose, two trajectories involving TS1 and TS2 transitions states were investigated by exploring the ground state potential energy surface of [Co(NH_3_)_5_NO_2_]^2+^: the trajectory from the Co–NO_2_ to *endo*-Co-ONO complex and that from the *endo*-Co-ONO to *exo*-Co-ONO complex. The mechanistic study of the intramolecular conversion reveals that the energy barrier for the intramolecular conversion of *endo*-Co-ONO in Co–NO_2_ (∼39 kcal mol^−1^) is 3.5 times larger than that of the interconversion between *endo*-Co-ONO and *exo*-Co-ONO. Our TS1 activation energy barrier carried out at ωB97XD/6-31+G(d,p) is 9 kcal mol^−1^ above the activation energies computed at B3LYP/LanL2DZ (5 kcal mol^−1^ at B3LYP/LanL2DZp) by Ciofini *et al.*^33^

##### Relative free energies

The relative free energies, enthalpies and entropies of the ground and transition states of the Co(NH_3_)_5_NO_2_^2+^ isomers calculated at the ωB97XD/6-31+G(d,p) theory level provided in Table 5 suggest that a direct transformation from nitro to *exo*-Co-ONO required more energy than that required for the transformation *via* the intermediate *endo*-Co-ONO. The energy difference between the activated complexes TS1 and TS3 is about 2.39 kcal mol^−1^. *endo*-Co-ONO appears to be more structured (low relative entropy) than Co–NO_2_ and *exo*-Co-ONO. The entropic energy differences can be associated with the strength of the hydrogen bonding in these systems. Overall, the entropy differences in these complexes are quite small ranging from 0.2 to 5 cal mol^−1^ and do not significantly affect the relative stabilities of Co–NO_2_ and Co–ONO complexes and their transition states. Because of the large energy of the TS2 transition-state, the molecular conversion between nitro and *endo*-nitrito is predicted to be the slow step of the reaction and may determine the rate of the intramolecular conversion between the Co–NO_2_ and *exo*-Co-ONO complexes. The most likely reaction mechanism involves starting from Co–NO_2_ and then transforming into the *endo*-Co-ONO intermediate and finally into *exo*-Co-ONO:

6 Co(NH_3_)_5_NO_2_^2+^ → [Co(NH_3_)_5_NO_2_^2+^]^≠^ (TS1) → *endo*-Co(NH_3_)_5_ONO^2+^

7 *endo*-Co(NH_3_)_5_ONO^2+^→[Co(NH_3_)_5_NO_2_^2+^]^≠^ (TS2) → *exo*-Co(NH_3_)_5_ONO^2+^

**Fig. 6:**
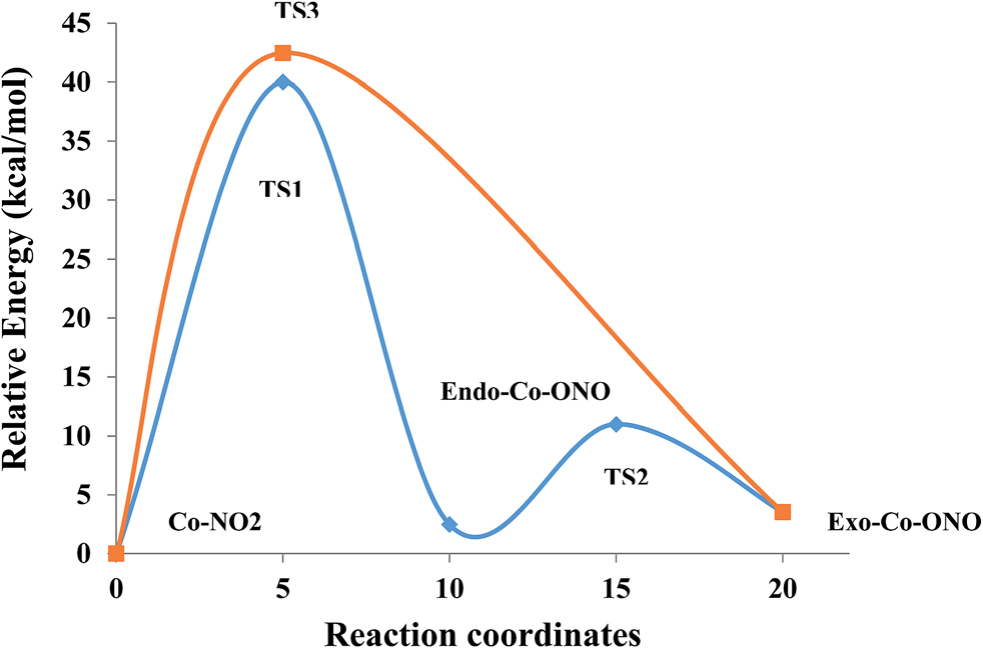
Reaction pathway of the conversion of the Co–NO_2_ into *exo*-Co–ONO at ωB97XD/6-31+G(d,p) in gase phase.

The pathway (1) proceeds through the higher-energy transition state (TS1), but yields the lower-energy nitrito conformer (*endo*-Co-ONO) (Fig. 6 and S5†). The second pathway (2) leads through the lower-energy transition (TS2), but yields a relatively higher-energy nitrito conformer (*exo*-Co-ONO). Therefore, the pathways (1) and (2) of the interconversion of Co–NO_2_ in *exo*-Co-ONO can be assumed to be respectively, the thermodynamically-favored and the kinetically-favored pathways (Fig. 6). TS4 and chelate structure (Fig. 1e) were also proposed as possible transition states.^11^ Our calculations show that the former (TS4, i95 cm^−1^) requires ∼233 kcal mol^−1^, while the chelate structure possesses several imaginary frequencies and relaxes towards TS2. However, the possibility of the reaction occurring *via* the TS4 state at standard conditions in gas phase is very low as the activation energy is too high. The reorientation of the NO_2_ ligand slightly changes the magnitude of the dipole moments from 10D in Co–NO_2_ to 11D in *exo*-Co–ONO (10D in *endo*-Co–ONO). With regards to the relative stabilities of linkage isomers, our results agree with previously reported experimental results,^17,18^ and these can justify the reorientation of the NO_2_ moiety in the [Co(NH_3_)_5_NO_2_]ClNO_3_ isomer upon UV irradiation to form the *exo*-[Co(NH_3_)_5_ONO]ClNO_3_ isomer and the slow conversion of the less stable *exo*-[Co(NH_3_)_5_ONO]ClNO_3_ complex into [Co(NH_3_)_5_NO_2_]ClNO_3_ in the dark. The fast conversion of *endo*-Co–ONO in the *exo*-Co–ONO conformer explains why the former has never been observed experimentally.

#### 3.2.3 Chemical bonding analysis

**Fig. 7:**
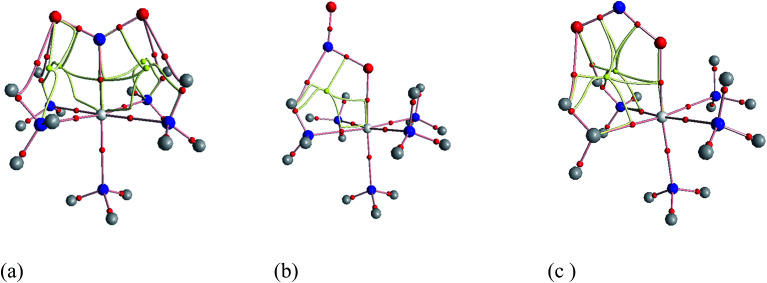
Molecular graphs (set of bond paths and critical points) of (a) Co–NO_2_ and (b–c) Co–ONO complexes obtained from AIM analysis of ωB97XD/6-31+G(d,p) wave function. Color code: large spheres (red: O, blue: N, grey: H, white: Co) and small spheres (red: bond critical points, and yellow: ring critical point).

In this section, we focus our analysis on the hydrogen bonding properties of the nitro and nitrito groups in Co–NO_2_ and Co–ONO complexes, respectively, in an attempt to support their rank order of stabilities. Fig. 7 depicts the molecular graphs obtained from atoms in molecules (AIM)^86^ analysis of Co–NO_2_ and Co–ONO complexes. The molecular graphs of these complexes have bond critical points (bcps) between H and ONO that clearly show that nitro and nitrito are interconnected with ammonia through O⋯H–N and N⋯H–N intramolecular hydrogen bonds (IHBs), respectively. The electron densities and their Laplacians at different bcps in the three Co-complexes are given in Table 6. The negative values of the Laplacians along N⋯H–N, and O⋯H–N bonds indirectly indicate that electrons are concentrated in these inter-nuclear regions. The O21⋯H12–N19 and O21⋯N19 bond lengths in *endo*-Co–ONO are 2.01 and 2.86 Å, respectively, leading to the possibility of competing O⋯N and O⋯H intramolecular interactions. The O21⋯N19 bond length in *endo*-Co–ONO lies in the same range as the N⋯O bond length (less than 3 Å) observed in crystals where nitro groups were arranged perpendicular to each other.^87^ The N⋯O bond is weaker than N⋯H and O⋯H bonds owing to the repulsion between lone pairs.^88^ The total NBO charge transfer energies^89,90^ from lone pairs (LPs) of N and O of ONO atoms group to the N–H σ*-antibonding orbitals are estimated to be about 10.49, 15.19, and 17.24 kcal mol^−1^ in *exo*-Co–ONO, Co–NO_2_, and *endo*-Co–ONO, respectively (Table 6); this indicates that intramolecular hydrogen bond strength increases in the following order: *exo*-nitrito < nitro < *endo*-nitro. The Co–O bond (NBO energy ∼ −1.00 kcal mol^−1^) is slightly more stable than the Co–N bond (NBO energy of −0.97 kcal mol^−1^). The stabilization energy *E*(2) associated with electron delocalization arising from the donor LPs of Co (d orbitals) to the acceptor Pi* of NO_2_ is estimated about 7.01 kcal mol^−1^ while that arising from Co to Pi* ONO amount to be about 1 kcal mol^−1^ (0.93 kcal mol^−1^ in *exo*-Co–ONO and 0.68 in *endo*-Co–ONO) computed at B3LYP/6-31G(d). This result shows that NO_2_ is a good pi* acceptor than ONO and is more favorable to pi*-back-bonding than the nitrito. Hence, the nitrito appears as a weaker pi electron acceptor than nitro. The latter can also explain the extra-stability of Co–NO_2_ complex compare to Co–ONO complexes.

**Table 6:** N⋯H–N and O⋯H–N NBO second-order perturbation energies *E*(2) (in kcal mol^−1^) and electron density (ρ) at the bcp and its Laplacian (∇^2^*ρ*) in parenthesis computed at ωB97XD/6-31+G(d,p)

Isomers	H Charge transfer	NBO E(2)	ρ(∇^2^ρ)
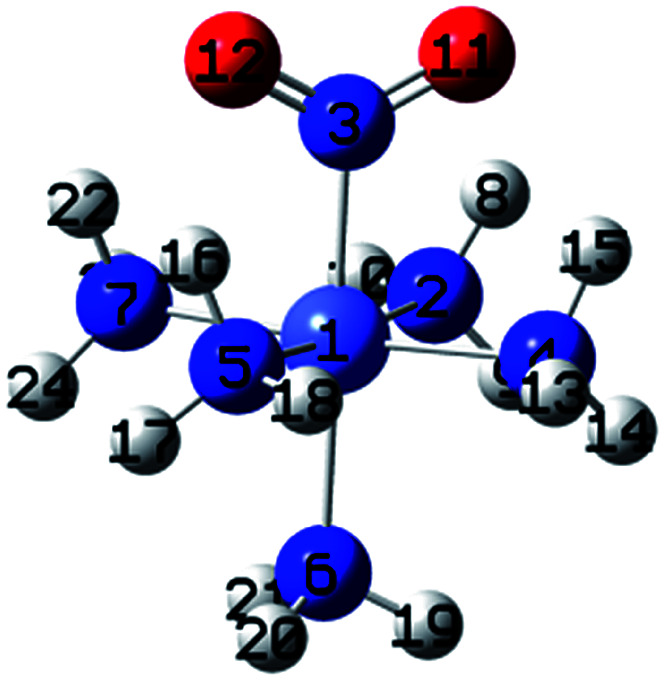	O12(LP) → N7–H22(σ*)	5.66	0.020(−0.018)
	O12(LP) → N5–H16(σ*)	3.92	0.017(−0.016)
	O11(LP) → N2–H8(σ*)	2.51	0.016(−0.016)
	O11(LP) → N4–H15(σ*)	3.10	0.019(−0.018)
	Total *E*(2)	15.19	
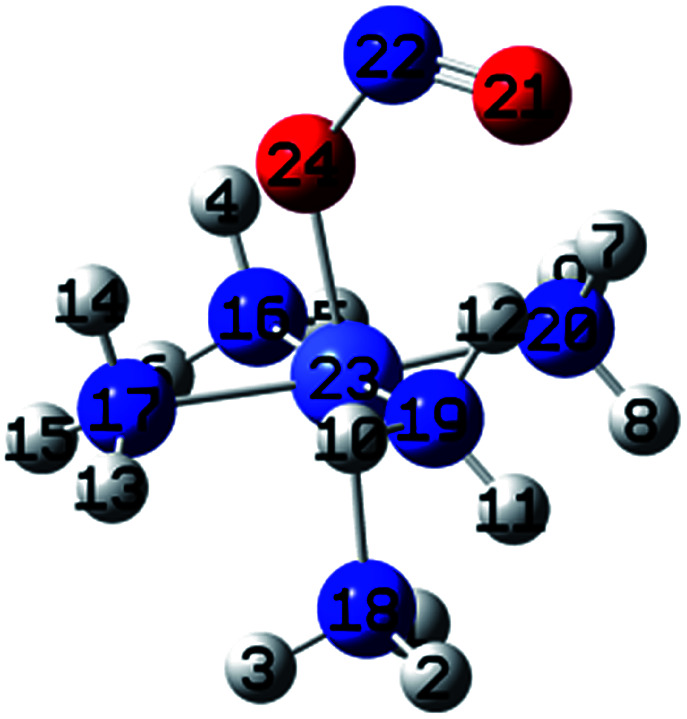	O21(LP) → N19–H12(σ*)	7.96	0.024(−0.018)
	O21(LP) → N7–H20(σ*)	7.06	0.022(−0.018)
	O24(LP) → N16–H4(σ*)	0.99	—
	O24(LP) → N17–H14(σ*)	1.23	—
	Total *E*(2)	17.24	
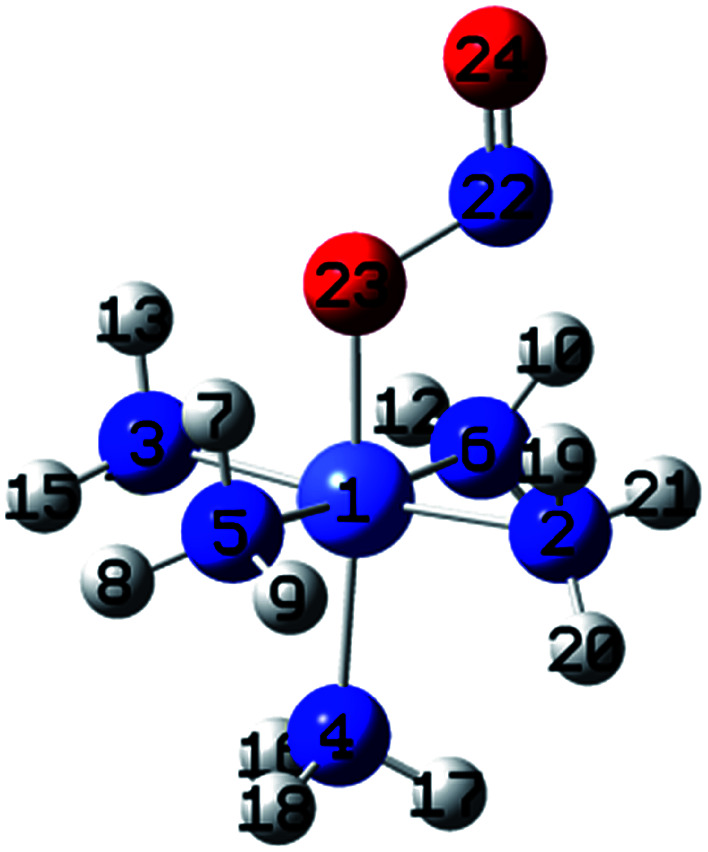	N22(LP) → N6–H10(σ *)	7.74	0.022(−0.018)
	O23(LP) → N5–H7(σ*)	1.24	—
	O23(LP) → N3–H13(σ*)	1.51	—
	Total *E*(2)	10.49	

**Fig. 8:**
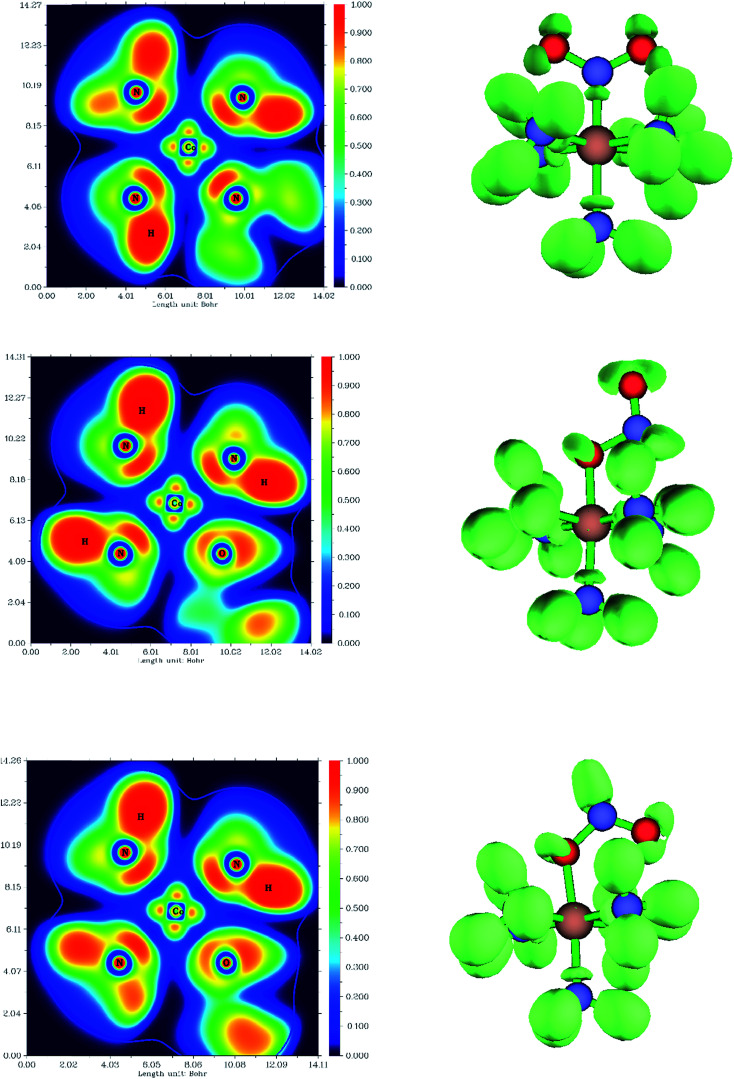
Electron localized function (ELF) in 2D (left) and 3D (right) of *endo*-Co–ONO.

**Fig. 9:**
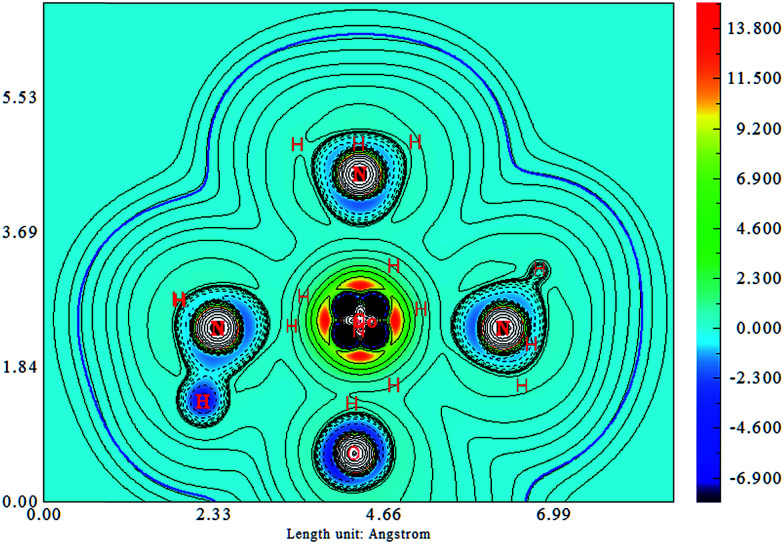
Laplacian in endo-Co–ONO.

The difference in the electronic structure between Co–NO_2_, *endo*-Co–ONO and *exo*-Co–ONO is shown in Table S6.† The N–O Mayer bond order and electrons occupancies of Co–NO_2_ and Co–ONO complexes are given in Table 7. The two N–O bonds in nitro have a 1.50 Mayer bond order, whereas in *endo*-Co–ONO, they are about 1.16 and 1.67 and are nearly 1.91 and 0.98 in *exo*-Co–ONO. These computed bond orders show that the electron density charge is delocalized over the two N–O bonds in Co–NO_2_ giving both of them a partial double bond character, while the electron delocalization is diminishing in Co–ONO. The electronic density delocalization over N–O bonds diminishes the most when nitro is converted in *exo*-Co–ONO. These findings suggest that the electron resonance in these complexes assists intramolecular hydrogen bonds and can justify the ranking relative stabilities of isomers. The electrostatic attraction between the Co and O atoms was proposed as driving force of the linkage isomerization due to the fact that the O atoms are rich in electrons than N in NO_2_ ligand.^91,92^Fig. 8 shows the ELF isosurfaces of nitro and nitrito complexes and their cut planes. The topologies of the molecular electron densities are similar, but a small difference can be noticed around ONO and NO_2_ (Table S7†). The N–H bond is of polar covalent type, while the bonds formed between Co and NH_3_ are of dative type, as indicated by the orientation of the lone pairs of electrons located on N. The ELF and electron density Laplacian cut planes (Fig. 8 and 9) clearly indicate the lone pair region of nitrogen and electron deficiency in Co that enable the NH_3_ donor ligand to form a dative bond.

**Table 7:** Mayer bond order of some selected bonds of nitro and nitrito-complexes computed at B3LYP/SVP (NBO occupancies at B3LYP/6-31G(d))

Bonds	*Endo*	*Exo*	Nitro
H–N	0.95(1.99)	0.95 (1.99)	0.96(1.99)
H_3_N–Co	0.66(1.97)	0.66 (1.97)	0.67(1.97)
Co–O	0.68(1.93)	0.83 (1.92)	—
N–Co	—	—	0.51(1.93)
O–N	1.67(1.99, 1.99)	1.91(2.00, 1.99)	1.50(1.99, 1.99)
N–O^a^	1.16(1.98)	0.98(1.99)	1.50(1.99)
LP (O)	(1.97, 1.85)	(1.99, 1.82)	(1.97, 1.84, 1.44)
LP(O)^a^	(1.92, 1.70)	(1.93, 1.77)	(1.97, 1.84)
LP(N)^b^	(1.91)	(1.91)	—
LP(Co)	(1.98, 1.97, 1.97)	(1.97, 1.97, 1.97)	(1.98, 1.94, 1.97)

a O bound to Co in *endo* and *exo*-nitrito complexes.

b LPs of N of nitro or nitrito.

## 4. Conclusion

The nitro–nitrito isomerization in Co(NH_3_)_5_NO_2_^2+^ linkage isomers was investigated from a theoretical perspective using quantum chemical calculations at ωB97XD/6-31+G(d,p), emphasizing on the structural, thermodynamic, and chemical bonding properties of the isomers. This isomerization is fundamental for a better understanding of the photo-salient effect in [(NH_3_)_5_CoNO_2_]ClNO_3_. The Bader theory based on the partitioning of electron density, the electron localization function, and natural bond orbitals were used for chemical bonding analysis. Our computational study led to the following conclusions:

(a) The nitrito/*exo*-nitrito isomerization reaction is predicted to occur *via* the following reaction pathway:

8 Co(NH_3_)_5_NO_2_^2+^ → [Co(NH_3_)_5_NO_2_^2+^]^≠^ (TS1) → *endo*-Co(NH_3_)_5_ONO^2+^

(b) The intramolecular conversion on the ground-state potential energy surface of Co–NO_2_ between *endo*-Co–ONO and *exo*-Co–ONO complexes is kinetically controlled and that leading to the Co–NO_2_ complex formation is thermodynamically controlled.

(c) O⋯H–N and N⋯H–N intramolecular hydrogen bonds, orientation of the atoms of the ONO group, and the difference in Co–N and Co–O bond energies are identified as key factors determining the relative stabilities of the Co–NO_2_ and Co–ONO linkage isomers.

(d) Co(NH_3_)_6_^3+^ is less stable compared to Co(NH_3_)_5_NO_2_^2+^ and undergoes a slight geometry distortion from *D*_3d_ to *D*_3_ characterized by a PJTE of 741 cm^−1^ at ωB97XD/6-31+G(d,p) (750 cm^−1^ at CCSD(T)/6-31+G(d,p)). It is worth noting that the hydrogen can freely rotate to keep the high octahedral symmetry.

The photo-isomerization of the [Co(NH_3_)_5_NO_2_]^2+^ complex is currently under investigation in our lab to determine the role of conical intersection and excited states in the UV-initiated intramolecular conversion of [Co(NH_3_)_5_NO_2_]NO_3_Cl.

## Conflicts of interest

The authors declare that they have no conflict of interest.

## Supplementary Material

RA-008-C7RA11603A-s001
