# Supplementary material for: Theoretical investigation on the ground state properties of the hexaamminecobalt(iii) and nitro–nitrito linkage isomerism in pentaamminecobalt(iii) in vacuo
Source: RSC Adv. 2018 Jan 16;8(6):3328–42. doi: 10.1039/c7ra11603a (PMC9077707; doi:10.1039/c7ra11603a)
Supplement: RA-008-C7RA11603A-s001 [file RA-008-C7RA11603A-s001.pdf]

Electronic Supplementary Material (ESI) for RSC Advances.
This journal is © The Royal Society of Chemistry 2018

Theoretical investigation on the ground state properties of the hexaamminecobalt(III) and nitro-nitrito linkage isomerism in pentaamminecobalt(III) *in vacuo*

Jules Tshishimbi Muya[a], Hoeil Chung[a*] and Sang Uck Lee[b,c*]*

[a]Hanyang University, Department of Chemistry, Seoul, South Korea

[b]Hanyang University, Department of Chemical & Molecular Engineering, Sangnok-gu, Ansan 426-791 Korea

[c]Hanyang University, Department of Bionanotechnology, Sangnok-gu, Ansan 426-791 Korea

Manuscript to be submitted to RSC Advances

Electronic supplementary information

Correspondent author:

Hoeil Chung

hoeil@hanyang.ac.kr

Version December 23, 2017

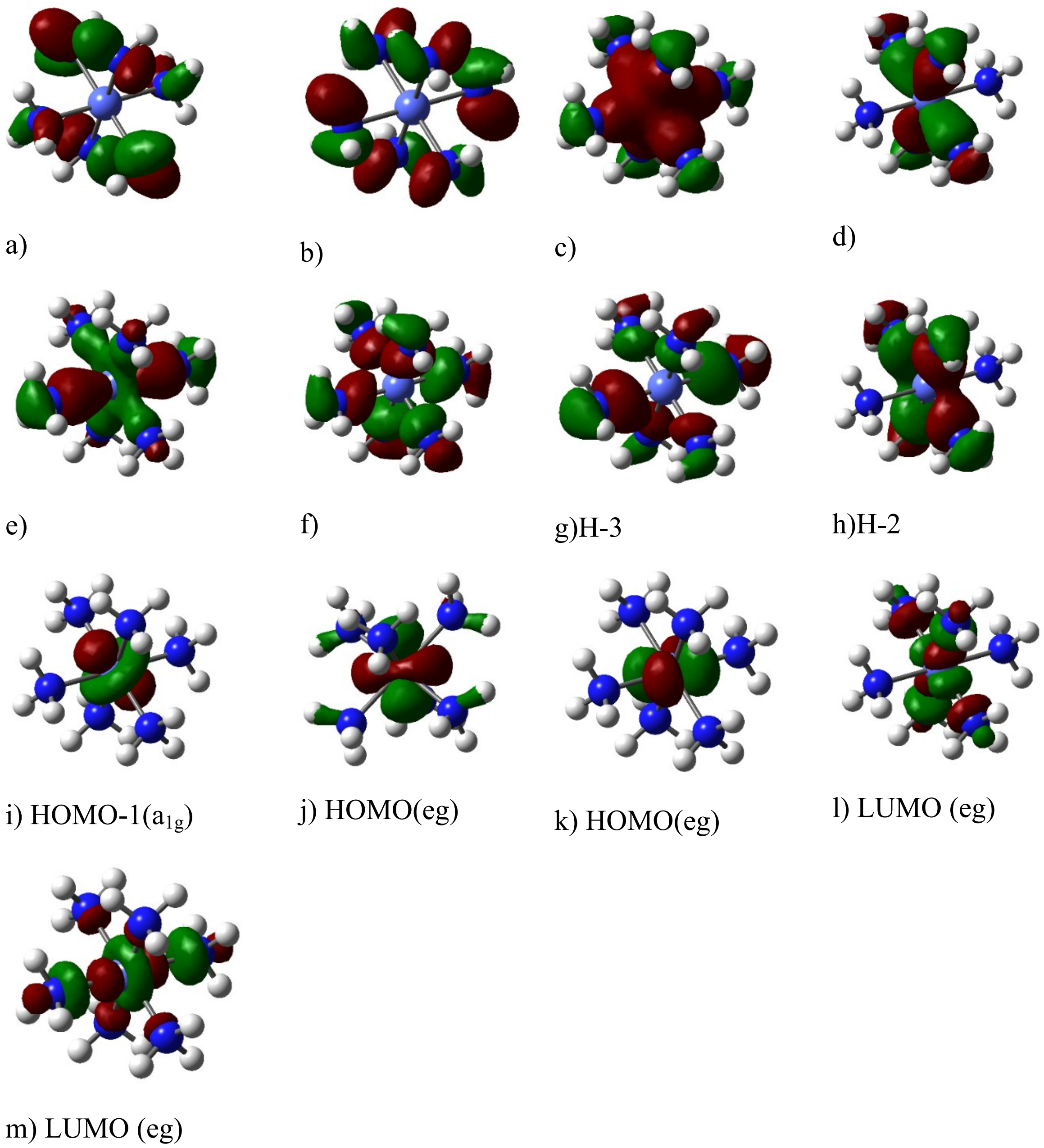

Figure S1. Selected valence molecular orbitals of D_{3d}-$[Co(NH_3)_6]^{3+}$ computed at wB97XD/6-31+G(d,p)

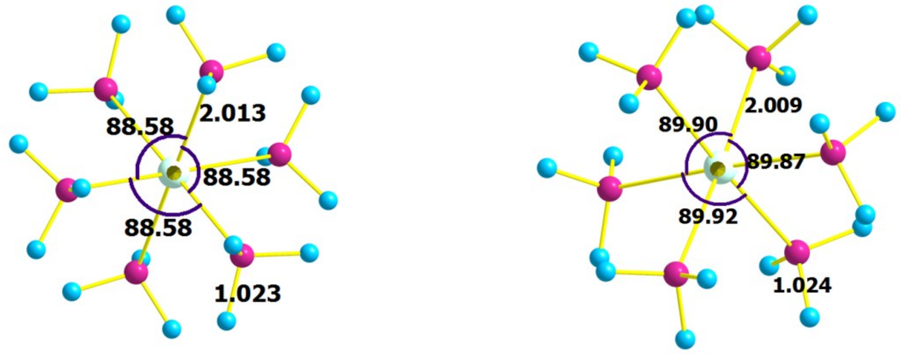

a) b)

Figure S2. Optimized geometries of D_{3d} (a) and D_3 (b) at wB97XD/6-31+G(d,p)

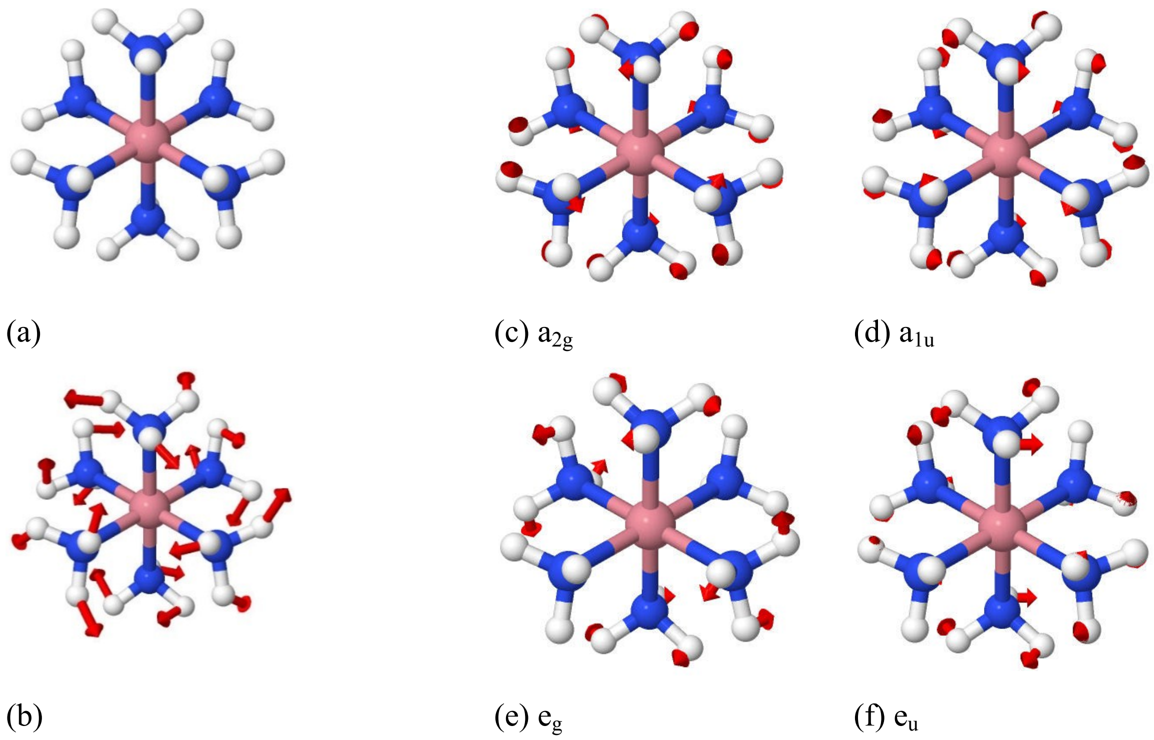

Figure S3. (a) Equilibrium geometry of $Co(NH_3)_6^{3+}$ in D_{3d} symmetry, (b) distortion vector from D_{3d} to D_3, and imaginary vibrational modes of a_{2g} (c), a_{1u} (d), e_g (e) and e_u (f) symmetry

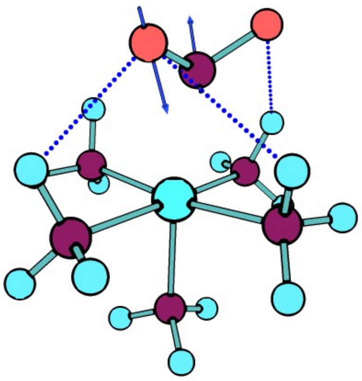

TS1 (deformation)
Nitro-→ endo-nitrito

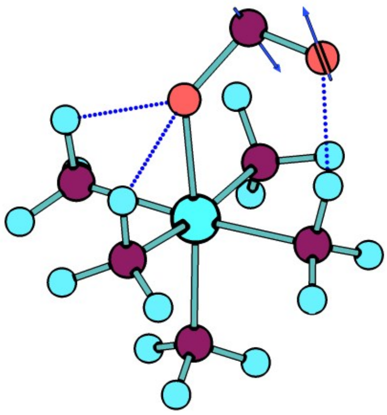

TS2 (deformation)
Endo-nitrito-→ exo-nitrito)

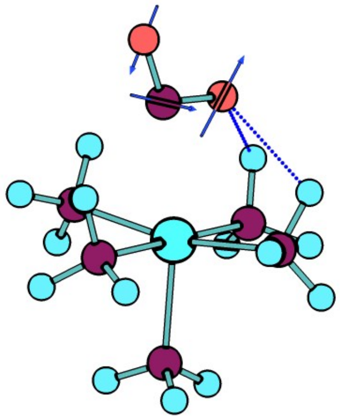

TS3 (rotation of NO_2)
(Nitro-→ exo-nitrito)

Figure S4. Imaginary vibrational modes of TS1-3

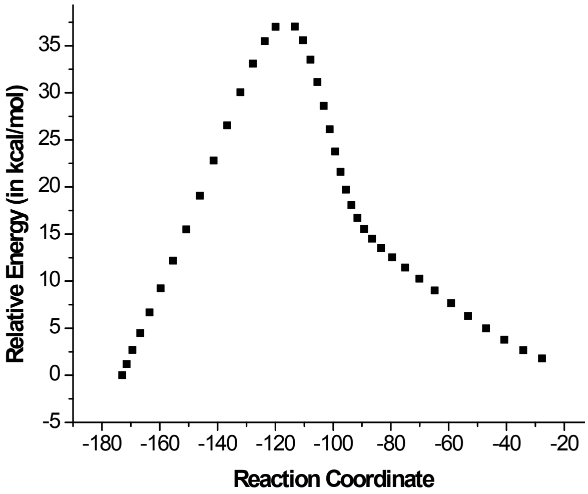

Figure S5. Intrinsic reaction coordinates plot of the intramolecular conversion between nitro isomer and endo-nitrito intermediate via **TS1** computed at wB97XD/6-31+G(d,p)

Table S1. Energies of valence molecular orbitals in Hartrees of D_3 and D_{3d}-$[Co(NH_3)_6]^{3+}$ computed at B3LYP/TZVP

D_{3d}	Energies	D_3	Energies
$6a_{2u}$	-0.8392	12e	-0.8355
$7e_u$	-0.8345	$7a_2$	-0.8354
$8a_{1g}$	-0.8007	$9a_1$	-0.7992
$6e_g$	*-0.7998*	*13e*	*-0.7988*
$7e_g$	-0.5700	14e	-0.5690
$9a_{1g}$	-0.4585	$10a_1$	-0.4572
$7a_{2u}$	-0.4091	15e	-0.4049
$8e_u$	-0.4039	$8a_2$	-0.4039

The HOMO is in italic

Table S2. Relative energies of the D_3, S_6 and C_{2h} epikernels with respect to D3 geometries computed at B3LYP, ωB97XD and OPBE with ccpVtz.

	B3LYP	ωB97XD	OPBE
D_3	-1.52	-1.78	-1.64
S_6	-1.18	-1.35	-1.24
C_{2h}	-1.16	-1.32	-1.21

Table S3. Imaginary frequencies of $[Co(NH)_3]_6^{3+}$ in D_{3d} and C_{2h} and its lowest real frequencies of D3 and S6 geometries computed at B3LYP, ωB97XD and OPBE with ccpVtz.

	B3LYP	ωB97XD	OPBE
D_{3d}	i132(a1u)+i128(a2g) +i55(eg)+i19(eu)	i145(a1u)+i141(a2g) +i69(eg)+i33(eu)	i135(a1u)+i131(a2g) +i49(eg)
D_3	80 (e)	81 (e)	84(e)
S_6	40 (eu)	49 (eu)	42 (eu)
C_{2h}	i61(au)+i40(bg)	i58(au)+i30(bg)	i65(au)+i38(bg)

Table. S4. Comparison of bond lengths and angles between X-ray structure and calculated geometries of Co-NO2, exo-Co-ONO, endo-Co-ONO and TS complexes computed at wB97XD/6-31+G(d,p) and B3LYP/LanL2DZ[] (values of bond lengths in Å and angles in degrees).

	nitro			nitrito			Endo-	TS1	
parameters	This work	Ciofini work[78]	Exp[79]	This work	Ciofini work[78]	Exp[80]	This work	This work	Ciofini work[78]
Co-N1	1.924	1.973	1.921	2.776	2.875		2.930	2.269	2.382
Co-N2	1.997	2.020	1.978	1.993	2.014	1.913	1.992	2.008	2.006
Co-N3	2.064	2.062	1.976	2.024	2.029	1.948	2.031	1.979	2.031
Co-N4	1.995	2.019	1.978	1.993	2.013	1.968	1.998	1.997	2.033
Co-N5	1.999	2.021	1.978	1.997	2.012	1.952	1.999	1.977	2.005
Co-N6	1.997	2.019	1.978	1.988	2.015	1.954	1.990	1.994	2.004
Co-O2	2.719	2.804	-	1.858	1.893	1.927	1.891	2.346	2.409
N1-O2	1.221	1.272	1.161	1.381	1.463	1.244	1.318	1.272	1.344

N1-O3	1.221	1.272	1.161	1.175	1.215	1.037	1.203	1.217	1.257
O2-N1-Co	117.89	117.7	123.0						
O2-N1-O3	124.4	124.4	113.9	112.3	112.5	125.3	116.7	118.8	118.3
Co-O2-N1				117.2	117.3	131.3	131.0		

[78] I. Ciofini, C. Adamo, JPHYSChemA 2001, 105, 1086-1092

[79] F.A Cotton, W.T Edwards, Acta Crystallogr. B 1968, 24, 474

[80] I. Grenthe, E. Nordin, Inorg. Chem. 1979, 18, 1869

Table S5. Relative energies (RE in kcal/mol) of different isomers and transition states, HOMO-LUMO gap energies (H-L in eV) of Co-NO_2 and Co-ONO computed using different methods at 6-31+G(d,p). TS1 and TS2 are transition states

Methods	RE(exo)	RE(endo)	RE(TS1)	RE(TS2)	H-L
CCSD(T)(a)	2.00	1.12	43.78	10.76	13.64
MP2	3.98	4.81	40.90	12.59	12.95
B3LYP	4.43	2.25	40.73	13.40	4.46
B3LYP-D3	4.42	1.83	-	5.64	4.52
M062X	-0.53	-2.77	36.76	7.00	7.65
wB97XD	3.39	1.32	40.89	12.70	8.68

(a) CCSD(T)/6-31G(d) single point calculation from MP2/6-31+G(d,p) optimized geometry

Table S6. Energies of valence orbitals in Hartrees of the nitro/nitrito linkage isomers computed at wB97XD/6-31+G(d,p)

Orbitals	Co-NO_2	exo-Co-ONO	endo-Co-ONO
L+3	-0.24467	-0.24246	-0.24311
L+2	-0.26644	-0.24388	-0.25377
L+1	-0.31722	-0.30936	-0.31871
L	-0.31968	-0.31741	-0.31964
H	-0.63876	-0.59813	-0.61054
H-1	-0.66656	-0.65652	-0.67549
H-2	-0.67931	-0.68464	-0.68020
H-3	-0.72491	-0.72708	-0.72869
H-4	-0.72828	-0.72858	-0.73044
H-5	-0.73013	-0.73434	-0.73698
H-6	-0.76630	-0.76118	-0.76295

The HOMO (H) and LUMO (L) are in italic

Table S7. Topological properties of the electron density of Co-NO_2 and Co-ONO : electron densities and their Laplacian in parenthesis at different bond critical points in Co-complexes computed at wB97XD/6-31+G(d,p).

Bonds	Co-NO_2	Exo-Co-ONO	Endo-Co-ONO
Co-NH_3	0.088(-0.108)	0.090(-0.109)	0.090(-0.107)
Co-N	0.118(-0.106)	-	-
Co-O	-	0.116(-0.154)	0.104(-0.136)
ON-O	0.501(0.263)	0.561(0.392)	0.523(0.339)
(Co)O-N	0.499(0.261)	0.340(0.102)	0.399(0.163)
N-H	0.331(0.448)	0.329(0.445)	0.331(0.448)
N…H		0.022(-0.018)	-
NO…H	0.019(-0.018)	-	0.022(-0.017)
O…N	-	-	

Gaussian09 outputs of D_3 and D_{3d}-$Co(NH_3)_6^{3+}$ calculations at CCSD(T) and BD(T) levels

(Enter /usr/local/gaussian/g09D01/g09/l9999.exe)

1\1\GINC-QUANTUM33\SP\RCCSD(T)-FC\6-31+G(d,p)\Co1H18N6(3+)\JMUYA\09-Ma
y-2016\0\\#P ccsd-t/6-31+G(d,p) symm=loose\\title\\3,1\Co,0,0.,0.,0.\N
,0,1.390677702,0.85644291,-1.156462665\N,0,0.046362466,-1.632583673,-1
.156462665\N,0,-1.437040168,0.776140763,-1.156462665\N,0,-1.437040168,
-0.776140763,1.156462665\N,0,0.046362466,1.632583673,1.156462665\N,0,1
.390677702,-0.85644291,1.156462665\H,0,1.52323731,0.418728808,-2.07316
8164\H,0,1.190998338,1.836474168,-1.378596498\H,0,2.334081969,0.887612
993,-0.758916332\H,0,-0.39898887,-1.528526611,-2.073168164\H,0,0.99493
4114,-1.9496719,-1.378596498\H,0,-0.398345583,-2.465180776,-0.75891633
2\H,0,-2.185932451,0.113197732,-1.378596498\H,0,-1.935736385,1.5775677
83,-0.758916332\H,0,-1.12424844,1.109797802,-2.073168164\H,0,-1.935736
385,-1.577567783,0.758916332\H,0,-1.12424844,-1.109797802,2.073168164\
H,0,-2.185932451,-0.113197732,1.378596498\H,0,-0.398345583,2.465180776
,0.758916332\H,0,-0.39898887,1.528526611,2.073168164\H,0,0.994934114,1
.9496719,1.378596498\H,0,1.190998338,-1.836474168,1.378596498\H,0,2.33
4081969,-0.887612993,0.758916332\H,0,1.52323731,-0.418728808,2.0731681
64\\Version=ES64L-G09RevD.01\State=1-A1\HF=-1717.4954481\MP2=-1718.888
0972\MP3=-1718.9224761\MP4D=-1718.9601737\MP4DQ=-1718.9416767\MP4SDQ=-
1718.9808716\CCSD=-1718.9834351**CCSD(T)=-1719.0362302**\RMSD=2.646e-09\P
G=**D03** [O(Co1),X(H18N6)]\\@

HE THAT WALD REACHE THE SWEITE ROSE
SULD NOW AND THEN BE SCRATCHED WT THE SCHARPE BREERES.
-- PROVERBS AND REASONS OF THE YEAR 1585
AS REPRINTED IN PAISLEY MAGAZINE 1828.

Job cpu time: 0 days 8 hours 23 minutes 14.5 seconds.

File lengths (MBytes): RWF= 7975 Int= 0 D2E= 0 Chk= 4 Scr= 1

Normal termination of Gaussian 09 at Mon May 9 05:34:18 2016.

(Enter /usr/local/gaussian/g09D01/g09/l9999.exe)

1\1\GINC-QUANTUM04\SP\RCCSD(T)-FC\6-31+G(d,p)\Co1H18N6(3+)\JMUYA\09-Ma
y-2016\0\\#P ccsd-t/6-31+G(d,p) symm=loose\\title\\3,1\Co,0,0.,0.,0.\N
,0,0.,1.617737548,1.181828667\N,0,-1.401001814,-0.808868774,1.18182866
7\N,0,1.401001814,-0.808868774,1.181828667\N,0,0.,-1.617737548,-1.1818
28667\N,0,1.401001814,0.808868774,-1.181828667\N,0,-1.401001814,0.8088
68774,-1.181828667\H,0,0.,1.410828064,2.184536\H,0,0.807748578,2.23698
5549,1.068027\H,0,-0.807748578,2.236985549,1.068027\H,0,-1.221812944,-
0.705414032,2.184536\H,0,-2.341160602,-0.418961986,1.068027\H,0,-1.533
412024,-1.818023563,1.068027\H,0,1.533412024,-1.818023563,1.068027\H,0
,2.341160602,-0.418961986,1.068027\H,0,1.221812944,-0.705414032,2.1845
36\H,0,-0.807748578,-2.236985549,-1.068027\H,0,0.,-1.410828064,-2.1845
36\H,0,0.807748578,-2.236985549,-1.068027\H,0,2.341160602,0.418961986,
-1.068027\H,0,1.221812944,0.705414032,-2.184536\H,0,1.533412024,1.8180
23563,-1.068027\H,0,-2.341160602,0.418961986,-1.068027\H,0,-1.53341202
4,1.818023563,-1.068027\H,0,-1.221812944,0.705414032,-2.184536\\Versio
n=ES64L-G09RevD.01\State=1-A1G\HF=-1717.4924952\MP2=-1718.884245\MP3=-
1718.9189593\MP4D=-1718.9566107\MP4DQ=-1718.9381087\MP4SDQ=-1718.97730
2\CCSD=-1718.9800145**CCSD(T)=-1719.032816**\RMSD=2.525e-09\PG=**D03D** [O(Co
1),3SGD(H2N2),X(H12)]\\@

UPON JULIA'S CLOTHES

WHENAS IN SILKS MY JULIA GOES,
THEN, THEN, METHINKS, HOW SWEETLY FLOWS
THAT LIQUEFACTION OF HER CLOTHES.
NEXT, WHEN I CAST MINE EYES, AND SEE
THAT BRAVE VIBRATION, EACH WAY FREE,
O, HOW THAT GLITTERING TAKETH ME!

-- ROBERT HERRICK, 1648

Job cpu time: 0 days 3 hours 51 minutes 46.2 seconds.

File lengths (MBytes): RWF= 3988 Int= 0 D2E= 0 Chk= 4 Scr= 1

Normal termination of Gaussian 09 at Mon May 9 05:01:13 2016.

(Enter /usr/local/gaussian/g09D01/g09/l9999.exe)

1\1\GINC-QUANTUM28\SP\RBD(T,FC)\6-31+G(d,p)\Co1H18N6(3+)\JMUYA\09-Jun-
2016\0\\#P BD(T)/6-31+G(d,p) symm=loose\\title\\3,1\Co,0,0.,0.,0.\N,0,
0.,1.617737548,1.181828667\N,0,-1.401001814,-0.808868774,1.181828667\N
,0,1.401001814,-0.808868774,1.181828667\N,0,0.,-1.617737548,-1.1818286
67\N,0,1.401001814,0.808868774,-1.181828667\N,0,-1.401001814,0.8088687
74,-1.181828667\H,0,0.,1.410828064,2.184536\H,0,0.807748578,2.23698554
9,1.068027\H,0,-0.807748578,2.236985549,1.068027\H,0,-1.221812944,-0.7
05414032,2.184536\H,0,-2.341160602,-0.418961986,1.068027\H,0,-1.533412
024,-1.818023563,1.068027\H,0,1.533412024,-1.818023563,1.068027\H,0,2.
341160602,-0.418961986,1.068027\H,0,1.221812944,-0.705414032,2.184536\
H,0,-0.807748578,-2.236985549,-1.068027\H,0,0.,-1.410828064,-2.184536\
H,0,0.807748578,-2.236985549,-1.068027\H,0,2.341160602,0.418961986,-1.
068027\H,0,1.221812944,0.705414032,-2.184536\H,0,1.533412024,1.8180235
63,-1.068027\H,0,-2.341160602,0.418961986,-1.068027\H,0,-1.533412024,1
.818023563,-1.068027\H,0,-1.221812944,0.705414032,-2.184536\\Version=E
S64L-G09RevD.01\State=1-A1G\HF=-1717.4924952\MP2=-1718.884245\MP3=-171
8.9189593\MP4D=-1718.9566107\MP4DQ=-1718.9381087\BDRef=-1717.4328454\B
D=-1718.9713181**BD(T)=-1719.0320755**\RMSD=2.524e-09**PG=D03D** [O(Co1),3SG
D(H2N2),X(H12)]\\@

THEORY: SUPPOSITION WHICH HAS SCIENTIFIC BASIS,
BUT NOT EXPERIMENTALLY PROVEN.

FACT: A THEORY WHICH HAS BEEN PROVEN BY ENOUGH
MONEY TO PAY FOR THE EXPERIMENTS.

-- THE WIZARD OF ID

Job cpu time: 0 days 6 hours 25 minutes 5.2 seconds.

File lengths (MBytes): RWF=   6222 Int=   0 D2E=   0 Chk=   5 Scr=   1

Normal termination of Gaussian 09 at Thu Jun 9 02:40:38 2016.

Leave Link 601 at Thu Jun 9 03:08:20 2016, MaxMem= 1610612736 cpu: 1.0

(Enter /usr/local/gaussian/g09D01/g09/l9999.exe)

1\1\GINC-QUANTUM15\SP\RBD(T,FC)\6-31+G(d,p)\Co1H18N6(3+)\JMUYA\09-Jun-
2016\0\\#P BD(T)/6-31+G(d,p) symm=loose\\title\\3,1\Co,0,0.,0.,0.\N,0,
1.390677702,0.85644291,-1.156462665\N,0,0.046362466,-1.632583673,-1.15
6462665\N,0,-1.437040168,0.776140763,-1.156462665\N,0,-1.437040168,-0.
776140763,1.156462665\N,0,0.046362466,1.632583673,1.156462665\N,0,1.39
0677702,-0.85644291,1.156462665\H,0,1.52323731,0.418728808,-2.07316816
4\H,0,1.190998338,1.836474168,-1.378596498\H,0,2.334081969,0.887612993
,-0.758916332\H,0,-0.39898887,-1.528526611,-2.073168164\H,0,0.99493411
4,-1.9496719,-1.378596498\H,0,-0.398345583,-2.465180776,-0.758916332\H
,0,-2.185932451,0.113197732,-1.378596498\H,0,-1.935736385,1.577567783,
-0.758916332\H,0,-1.12424844,1.109797802,-2.073168164\H,0,-1.935736385
,-1.577567783,0.758916332\H,0,-1.12424844,-1.109797802,2.073168164\H,0
,-2.185932451,-0.113197732,1.378596498\H,0,-0.398345583,2.465180776,0.
758916332\H,0,-0.39898887,1.528526611,2.073168164\H,0,0.994934114,1.94
96719,1.378596498\H,0,1.190998338,-1.836474168,1.378596498\H,0,2.33408
1969,-0.887612993,0.758916332\H,0,1.52323731,-0.418728808,2.073168164\
\Version=ES64L-G09RevD.01\State=1-A1\HF=-1717.4954481\MP2=-1718.888097
2\MP3=-1718.9224761\MP4D=-1718.9601737\MP4DQ=-1718.9416767\BDRef=-1717
.4360641\BD=-1718.9747775**BD(T)=-1719.0355082**\RMSD=2.645e-09**PG=D03** [O
(Co1),X(H18N6)]\\@

GARBAGE IN, GARBAGE OUT

Job cpu time: 0 days 14 hours 46 minutes 29.9 seconds.

File lengths (MBytes): RWF= 11680 Int= 0 D2E= 0 Chk= 5 Scr= 1

Normal termination of Gaussian 09 at Thu Jun 9 03:08:22 2016.
